# Increased toll-like receptors and p53 levels regulate apoptosis and angiogenesis in non-muscle invasive bladder cancer: mechanism of action of P-MAPA biological response modifier

**DOI:** 10.1186/s12885-016-2474-z

**Published:** 2016-07-07

**Authors:** Patrick Vianna Garcia, Fábio Rodrigues Ferreira Seiva, Amanda Pocol Carniato, Wilson de Mello Júnior, Nelson Duran, Alda Maria Macedo, Alexandre Gabarra de Oliveira, Rok Romih, Iseu da Silva Nunes, Odilon da Silva Nunes, Wagner José Fávaro

**Affiliations:** Laboratory of Urogenital Carcinogenesis and Immunotherapy, Department of Structural and Functional Biology, University of Campinas (UNICAMP), P.O. BOX 6109, zip code 13083-865 Campinas, São Paulo Brazil; Institute of Biology, North of Parana State University (UENP), Bandeirantes, PR Brazil; Department of Anatomy, Institute of Biosciences, UNESP - Univ Estadual Paulista, Botucatu, SP Brazil; Farmabrasilis R&D Division, Campinas, SP Brazil; NanoBioss, Institute of Chemistry, University of Campinas (UNICAMP), Campinas, SP Brazil; Department of Internal Medicine, University of Campinas (UNICAMP), Campinas, SP Brazil; Department of Physical Education, São Paulo State University (UNESP), Rio Claro, SP Brazil; Institute of Cell Biology, Faculty of Medicine, University of Ljubljana, Ljubljana, Slovenia

**Keywords:** Bladder Cancer, Toll-like Receptor, p53, Immunotherapy, P-MAPA, Angiogenesis, *Bacillus Calmette–Guerin*

## Abstract

**Background:**

The new modalities for treating patients with non-muscle invasive bladder cancer (NMIBC) for whom BCG (*Bacillus Calmette-Guerin*) has failed or is contraindicated are recently increasing due to the development of new drugs. Although agents like mitomycin C and BCG are routinely used, there is a need for more potent and/or less-toxic agents. In this scenario, a new perspective is represented by P-MAPA (Protein Aggregate Magnesium-Ammonium Phospholinoleate-Palmitoleate Anhydride), developed by Farmabrasilis (non-profit research network). This study detailed and characterized the mechanisms of action of P-MAPA based on activation of mediators of Toll-like Receptors (TLRs) 2 and 4 signaling pathways and p53 in regulating angiogenesis and apoptosis in an animal model of NMIBC, as well as, compared these mechanisms with BCG treatment.

**Results:**

Our results demonstrated the activation of the immune system by BCG (MyD88-dependent pathway) resulted in increased inflammatory cytokines. However, P-MAPA intravesical immunotherapy led to distinct activation of TLRs 2 and 4-mediated innate immune system, resulting in increased interferons signaling pathway (TRIF-dependent pathway), which was more effective in the NMIBC treatment. Interferon signaling pathway activation induced by P-MAPA led to increase of iNOS protein levels, resulting in apoptosis and histopathological recovery. Additionally, P-MAPA immunotherapy increased wild-type p53 protein levels. The increased wild-type p53 protein levels were fundamental to NO-induced apoptosis and the up-regulation of BAX. Furthermore, interferon signaling pathway induction and increased p53 protein levels by P-MAPA led to important antitumor effects, not only suppressing abnormal cell proliferation, but also by preventing continuous expansion of tumor mass through suppression of angiogenesis, which was characterized by decreased VEGF and increased endostatin protein levels.

**Conclusions:**

Thus, P-MAPA immunotherapy could be considered an important therapeutic strategy for NMIBC, as well as, opens a new perspective for treatment of patients that are refractory or resistant to BCG intravesical therapy.

**Electronic supplementary material:**

The online version of this article (doi:10.1186/s12885-016-2474-z) contains supplementary material, which is available to authorized users.

## Background

Bladder cancer (BC) is the fourth most incidence tumor in men and the ninth in women, showing high morbidity and mortality rates [1, 2]. More than 70 % of BC is superficial (non-muscle invasive bladder cancer) and classified into 3 stages: pTis (flat carcinoma in situ), pTa (papillary carcinoma non-invasive) and pT1 (tumor invading mucosa or submucosa of the bladder wall) [3, 4]. Despite the prognosis associated with non-muscle invasive bladder tumours, almost 50 % of patients will experience recurrence of their disease within 4 years of their initial diagnosis, and 11 % will progress to muscle invasive disease [3].

The primary treatment for high-grade NMIBC is based on surgery by transurethral resection of bladder tumor (TURBT), followed by intravesical immunotherapy with Bacillus Calmette–Guerin (BCG) [5]. The response induced by BCG reflects induction of a T-helper type-1 (Th1) response to prevent recurrence and to reduce tumor progression [5–7]. However, BCG therapy shows several undesirable effects that are observed up to 90 % of patients, such as fever, chills, fatigue, irritative symptoms, haematuria and until major complications as sepsis and death [8, 9].

Based on this background, compounds activating the immune system, including vaccines, biological response modifiers and tumor environment modulators are, considered potential candidates for the development of new NMBIC treatments aiming to obtain greater therapeutic effect combined with lower toxicity. Toll-like receptors (TLRs) agonist compounds may represent a potential antitumor therapeutic approach, as these receptors are implicated in the pathogenesis of some tumors, including NMIBC [10–12]. TLRs play key roles in innate immunity and their activation can trigger two different responses in tumors: they stimulate immune system to attack tumor cells and/or eliminate the inhibitory machinery to the immune system [13–15]. TLRs signaling consist of two pathways: MyD88-dependent (canonical) and TRIF-dependent (non-canonical) pathways [13–15]. Except for TLR3, the MyD88-dependent pathway activates NF-kB and MAPK, resulting in inflammatory cytokines release, such as Tumor Necrosis Factor α (TNF-α) and interleukin-6 (IL-6) [13, 14]. Conversely, the TRIF-dependent pathway activates Interferon Regulatory Factor 3 (IRF-3) for the production of interferon [13–15]. TLR4 is the only receptor that uses the four adapter molecules (MyD88, TRIF, TRAM and TIRAP) in a signal cascade [13–15].

Most TLRs genes respond to *p53* via canonical as well as non-canonical promoter binding sites [16]. The p53 protein is responsible for cell cycle regulation, and it acts as tumor suppressor [16, 17]. Studies of response element promoter sequences targeted by *p53* suggest a general role for *p53* as a regulator of DNA damage and as a control of TLRs gene expression [16]. Furthermore, several studies suggested that antiangiogenic therapy is sensitive to p53 status in tumors, indicating an important role of p53 in the regulation of angiogenesis [18, 19].

Angiogenesis plays a fundamental role in initiation and progression in different tumors [20]. The vascular endothelial growth factor (VEGF) stimulates all aspects of endothelial function such as: proliferation, migration, production of nitric oxide (NO) and endothelial cell layer permeability [18, 20–22]. The angiogenesis inhibitors have been developed to target endothelial cells and blocking tumor blood supply [18, 23]. Endostatin is a potent endogenous inhibitor of angiogenesis and induces apoptosis in both endothelial cells and tumor cells [18, 19, 24].

Immunotherapy using compounds that act as TLR agonists could be a valuable approach for cancer treatment, whether used alone or in combination with existing therapies. Protein aggregate magnesium-ammonium phospholinoleate-palmitoleate anhydride (P-MAPA) a biopolymer isolated in the 70′s [25] and characterized in the years 90′s [26–28] currently under development by Farmabrasilis (a nonprofit research network) [29], has emerged as a potential candidate for intravesical therapy for NMIBC. P-MAPA is a biological response modifier obtained by fermentation from *Aspergillus oryzae* that demonstrates important antitumor effect in several animal models of cancer, including NMIBC [11, 12, 26–28]. Recent studies of our research group demonstrated that P-MAPA modulates TLR 2 and 4 in both infectious diseases and cancer [11, 12, 30].

The strategy of research and development of the drug P-MAPA is based in the concept of open source model, with the researchers linked by a virtual research network [29]. A complementary strategy adopted by Farmabrasilis aims to booster the production of data to accelerate the development of the compound as drug candidate for cancer, including NMIBC, involves the selection of compounds already in clinical use, and when available, compounds equally able to act together with P-MAPA, such as BCG, used in parallel or in conjunction with experiments in vivo. The use of immunomodulatory compounds already known against NMIBC with mechanisms of action partially elucidated, such as BCG, in comparative studies with P-MAPA using the same animal model, may facilitate the visualization of commonalities, as well as the differences in the mechanisms of action. Of note, these data may also be relevant to understand the mode of action of P-MAPA, aiming the elaboration of new strategies focusing the future use of the compound for treatment of some conditions that emerge in the treatment of NMIBC, such as BCG refractory and BCG relapsing diseases.

Thus, this study presents the first comprehensive view of the mechanisms of a potential therapeutic agent for NMIBC, P-MAPA biological response modifier, based on activation of mediators of TLRs 2, 4 and p53 signaling pathways in regulating the angiogenesis and apoptosis processes.

## Methods

### NMIBC induction and treatment

Forty female Fischer 344 rats, all 7 weeks old, were obtained from the Multidisciplinary Center for Biological Investigation (CEMIB) at University of Campinas (UNICAMP). For the experiments the protocol followed strictly the ethical principles in animal research (CEUA/IB/UNICAMP–protocol number: 2684-1). Before each intravesical catheterisation via a 22-gauge angiocatheter treatments, animals were anesthetized with 10 % ketamine (60 mg/kg, i.m.; Ceva Animal Health Ltda, São Paulo, Brazil) and 2 % xylazine (5 mg/kg, i.m.; Ceva Animal Health Ltda, São Paulo, Brazil). The animals remained anesthetized for approximately 45 min after catheterization to prevent spontaneous micturition. Ten control animals (CONTROL group) received 0.30 ml of 0.9 % physiological saline every other week for 14 weeks. Thirty animals received 1.5 mg/Kg of n-methyl-n-nitrosourea (MNU) dissolved in 0.30 mL of sodium citrate (1 M pH 6.0); each intravesically every other week for 8 weeks [11, 12]. Two weeks after the last dose of MNU, all animals were submitted to retrograde cystography and ultrasonography to evaluate the occurrence of tumor. Both negative and positive contrast cystography enabled the bladder wall, mucosal margin and lumen to be visualised. For positive or negative contrast cystographies, animals were submitted to intravesical catheterisation via a 22-gauge angiocatheter to drain all the urine from the bladder, instilled 0,3 mL of positive contrast medium or 0,3 mL of air (negative contrast) into the bladder until becomes slightly turgid (judged by palpation of the bladder through the abdominal wall) and taken lateral and ventrodorsal radiographs.

The ultrasounds were evaluated using a portable, software-controlled ultrasound system with a 10–5 MHz 38-mm linear array transducer.

**Fig. 1:**
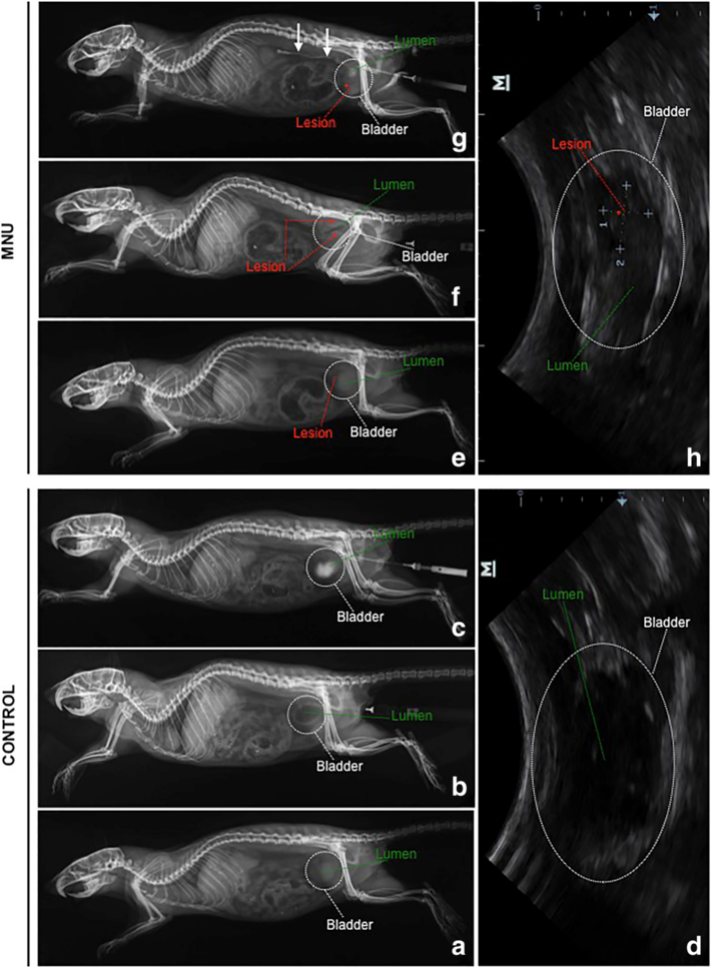
**a–h** Retrograde cystography and ultrasonography from CONTROL (**a, b, c, d**) and MNU (**e, f, g, h**) groups. Cystography without contrast (**a**), negative (**b**) and positive (**c**) contrast cystographies, and ultrasounds (**d**) showed no mass infiltrating the bladder walls, as well as, there were no vesicoureteral reflux and neither bladder filling defect. Cystography without contrast (**e**) and negative contrast cystography (**f**) showed a mass infiltrating the ventral, dorsal and cranial bladder walls (**asterisks**). Positive contrast cystography (**g**) demonstrated several bladder filling defects and vesicoureteral reflux unilateral (**arrows**). Ultrasound showed tumor (**asterisk**) infiltrating the bladder walls, tumor size: 1–3,9 mm, 2–5,5 mm

The animals from CONTROL group showed no mass infiltrating the bladder walls, as well as, there were no vesicoureteral reflux and neither bladder filling defect (Fig. 1a, b, c and d).

Negative contrast cystography and ultrasonography of urinary bladder from MNU group showed a mass (average tumor size 3,5 × 5,1 mm) infiltrating the ventral, dorsal and cranial bladder walls (Fig. 1e, f and h). Positive contrast cystography demonstrated several bladder filling defects and vesicoureteral reflux unilateral (Fig. 1g) in 80 % of animals and bilateral in 10 % of animals.

MNU treated animals were further divided into three groups (ten animals per group): the MNU group received 0.30 ml of 0.9 % physiological saline; the MNU-BCG group received 10^6^ CFU (40 mg) of BCG (Fundação Ataulpho de Paiva, Rio de Janeiro, RJ, Brazil); the MNU-P-MAPA group received 5 mg/kg dose of P-MAPA (Farmabrasilis, Campinas, SP, Brazil). All animals were treated every other week for 6 weeks. After the treatment, the animals were euthanized and their urinary bladder were collected and processed for histopathological, immunological and Western Blotting analysis.

### Histopathological analysis

Samples of urinary bladders were used (*n* = 5) of each group and fixed in Bouin solution for 12 h. Then, after the fixation, the fragments were washed in 70 % ethanol, and dehydrated in an ascending series of alcohols. Subsequently, the fragments were diaphanized in xylene for 2 h and embedded in the plastic polymer (Paraplast Plus, ST. Louis, MO, USA). Subsequently, the samples were cut on a rotary microtome *Slee CUT5062 RM 2165* (Slee Mainz, Mainz, Germany), 5 μm thick, stained with hematoxylin-eosin and photographed with a *Leica DM2500* photomicroscope (Leica, Munich, Germany). A senior uropathologist analyzed the urinary bladder lesions according to Health/World International Society of Urological Pathology Organization [4].

### Immunohistochemistry of toll-like receptor signaling pathway: (TLR2, TLR4, MyD88, IRF-3, IKK-α, BAX, NF-kB, iNOS, TNF-α, TRIF, IFN-γ, IL-6) and proliferation (Ki-67) in NMIBC

The same samples as for histopathological analysis were used for immunolabelings. They were cut into 6 μm thick sections and antigen retrieval was performed either by different protocols. Following that, the sections were incubated in 0.3 % H2O2 to block endogenous peroxidase, and nonspecific binding was blocked by incubating the sections in blocking solution at room temperature. The primary antibodies were: rabbit polyclonal anti-TLR2 (251110, Abbiotec, San Diego, USA; 1:100), rabbit polyclonal anti-TLR4 (251111, Abbiotec, San Diego, USA; 1:100), rabbit polyclonal anti-MyD88 (ab2064; 1:75), rabbit polyclonal anti-IRF-3 (ab25950; 1:150), rabbit polyclonal anti-IKK-α (ab38515; 1:100), rabbit polyclonal anti-BAX (ab7977; 1:50), rabbit polyclonal anti-NF-kB (ab7970; 1:200), rabbit polyclonal anti-iNOS (ab15323; 1:75), rabbit polyclonal anti-TNF-α (ab6671; 1:150), rabbit polyclonal anti-TRIF (ab13810; 1:100), rabbit polyclonal anti-IL-6 (ab6672; all the above from Abcam, USA), mouse monoclonal anti-IFN-γ (507802, Biolegend, USA;1:50) and mouse monoclonal anti- Ki-67 (NCL-Ki67-MM1, Novocastra; Newcastle, United Kingdom; 1:50). Antibodies were diluted in 1 % BSA and applied to the sections overnight at 4 °C. Bound antibodies were detected with an *AdvanceTM HRP* kit (Dako Cytomation Inc., USA). Sections were lightly counterstained with Harris’ hematoxylin and photographed with a photomicroscope (*DM2500* Leica, Munich, Germany).

The immunohistochemistries were measured in five animals in each experimental group, the same samples as for histopathological analysis. Ten microscopic fields per animal were measured with 40·objective lens and corresponded to a total area of 92,500.8 μm^2^. TLR2, TLR4, MyD88, IRF-3, IKK-α, BAX, NF-kB, iNOS, TNF-α, TRIF, IFN-γ, IL-6 antibodies were scored semiquantitatively by recording percentage of only urothelial cells. At least 1,000 urothelial cells, for each group (200 urothelial cells per animal), were counted by the software LAS V 3.7 (Leica, Munich, Germany) while the examiner classified them as positive or negative cells. Thus, the percentage of labeled cells (PLC) was determined, according to the following equation:

$$ \mathrm{P}\mathrm{L}\mathrm{C}=\mathrm{number}\ \mathrm{of}\ \mathrm{labelled}\ \mathrm{cells}/\mathrm{total}\ \mathrm{counted}\ \mathrm{cells}\times 100\hbox{--} \mathrm{expressed}\ \mathrm{in}:\% $$

The PLC values were categorized into four scores as follows: 0, no immunoreactivity; 1, 1–35 % positive urothelial cells; 2, 36–70 % positive urothelial cells; 3, > 70 % positive urothelial cells. The software LAS V 3.7 (Leica, Munich, Germany) was used to quantify the intensity of brownish-color immunostaining. For each antibody, the same photomicrographs used for determining the PLC were considered. Ten randomized labeled nuclear and/or cytoplasmic regions from different urothelial cells were indicated, with the same-sized square (software LAS V 3.7). The average optical density (OD) of these areas was automatically calculated and represents the average of red, green, and blue color composition (RGB) per area of nucleus and/or cytoplasm analyzed, expressed in optical units per micrometer squared (ou/μm^2^). The same procedure was applied to obtain the background optical density (BOD) from an area without tissue or vascular space for each photomicrograph. A single area was enough, since the background was constant in each photomicrograph. The absolute white colour that corresponds to the maximum optical density (M_ax_OD) was composed by the totality of red, green, and blue; and black was the absence of these colors. Therefore, the optical density values calculated by the software make up a decreasing scale in which the high values correspond to the colours that are visually clear.

The equation below was used to calculate the digital immunostaining intensity (ITI_dig_) for each antibody, whose values make up an increasing scale, equalized by the BOD, proportionally to the optical density of absolute white:

$$ \mathbf{IT}{\mathbf{I}}_{\mathbf{dig}}={\mathbf{M}}_{\mathbf{ax}}\mathbf{O}\mathbf{D}-{\mathbf{M}}_{\mathbf{ax}}\mathbf{O}\mathbf{D}\times \mathbf{\sum}\mathbf{O}\mathbf{D}/\mathbf{\sum}\mathbf{BOD}\hbox{--} \mathbf{expressed}\ \mathbf{in}:\mathbf{o}\mathbf{u}/\boldsymbol{\upmu} {\mathbf{m}}^{\mathbf{2}} $$

The intensity of reactivity was recorded as: weak (1+, ITI_dig_ average = 49.3 μm^2^), moderate (2+, ITI_dig_ average = 71.3 μm^2^) and intense (3+, ITI_dig_ average = 95.1 μm^2^).

### Western blotting analysis of toll-like receptor signaling pathway and angiogenesis: TLR2, MyD88, IKK-α, NF-kB, TNF-α, IL-6, TLR4, TRIF, IRF-3, IFN-γ, iNOS, p53, vascular endothelial growth factor (VEGF), endostatin BAX and nod-like receptor 5 (NLRC5) in NMIBC

Samples of the urinary bladders were used (*n* = 5) of each group, weighed (average 200 mg) and homogenized in 50 μl/mg of RIPA lysis buffer (EMD Millipore Corporation, Billerica, MA, USA). Aliquots containing 70 μg of protein were separated by SDS-PAGE on 10 % or 12 % polyacrylamide gels under reducing conditions. After electrophoresis, the proteins were transferred to Hybond-ECL nitrocellulose membranes (Amersham, Pharmacia Biotech, Arlington Heights, IL., USA). The membranes were blocked with TBS-T containing 1 % BSA (bovine serum albumin) and incubated overnight at 4 °C with with primary rabbit polyclonal anti-TLR2 (ab13855; abcam, USA) polyclonal rabbit anti-MyD88 (ab2064; abcam, USA), polyclonal rabbit anti-IKK-α (ab38515; abcam, USA), polyclonal rabbit anti-NF-kB (ab7970; abcam, USA), polyclonal rabbit anti-TNF-α (ab6671; abcam, USA), polyclonal rabbit anti-IL-6 (ab6672; abcam, USA), mouse monoclonal anti-TLR4 (ab30667; abcam, USA), polyclonal rabbit anti-TRIF (ab13810; abcam, USA), polyclonal rabbit anti-IRF-3 (ab25950; abcam, USA), mouse monoclonal anti-IFN-γ (507802; Biolegend, USA), polyclonal rabbit anti-iNOS (ab15323; abcam, USA), mouse monoclonal anti-p53 (ab26; abcam, USA), monoclonal mouse anti-VEGF (sc-53462; Santa Cruz Biotechnology, USA), monoclonal mouse anti-Endostatin (ab64569; abcam, USA), polyclonal rabbit anti-BAX (ab7977; abcam, USA), polyclonal rabbit anti-NLRC5 (ab105411; abcam, USA) for diluted in 1 % BSA. The membranes were then incubated for 2 h with rabbit or mouse secondary HRP-conjugated antibodies (diluted 1:3,000 in 1 % BSA; Santa Cruz Biotechnology, Inc., Santa Cruz, CA, USA). Peroxidase activity was detected by incubation with a diaminobenzidine chromogen (Sigma Chemical Co., St Louis, USA). Western blots were run in duplicate, and urinary bladder samples were pooled from 5 animals per group for each repetition. The semi-quantitative densitometry (IOD – Integrated Optical Density) analysis of bands was conducted using NIH ImageJ 1.47v software (National Institute of Health, USA. Available in: http://rsb.info.nih.gov/ij/), followed by statistical analysis. β-actin was used as endogenous positive controls for standardization of the readings of band staining intensity. The results were expressed as the mean ± standard deviation of the ratio of each band’s intensity to β-actin band intensity [12].

### Determination of the proliferative index

Samples of the urinary bladders were randomly collected from 5 animals in each group, the same used for Ki-67 immunodetection and histopathology, and used for determination of the proliferative index. Ten fields were taken at random and measured per animal, resulting in 50 fields per group with an × 40 objective lens and the total number of Ki-67 staining positive cells was expressed as the percentage of these total cells, including luminal and basal epithelial cells. Sections were lightly counterstained with methyl green.

### Detection of apoptosis and determination of the apoptotic index

Samples of the urinary bladders from five animals in each group, the same used for immunodetection and histopathology, were processed for DNA fragmentation (TUNEL) by means of Terminal Deoxynucleotidyl Transferase (TdT), using the Kit FragEL™ DNA (Calbiochem, La Jolla, CA, USA). The apoptotic nuclei were identified using a diaminobenzidine chromogen mixture (Kit FragEL™ DNA). Ten microscopic fields were randomly taken and analyzed per sample, resulting in 50 fields per group, using a *Leica DM2500* (Leica, Munich, Germany) photomicroscope with a × 40 objective. Sections were lightly counterstained with methyl green. The apoptotic index was determined by dividing the number of apoptotic nuclei by the total number of nuclei found in the microscope field.

### Statistical analyses

Western Blotting, proliferative and apoptotic indexes and proliferation/apoptotic ratio (P/A) were statistically compared among the groups by analysis of variance followed by the Turkey’s test with the level of significance set at 1 %. Results were expressed as the mean ± standard deviation. Histopathological analyses were evaluated by proportion test. The difference between the two proportions was tested using test of proportion. For all analyses, a type-I error of 5 % was considered statistically significant.

## Conclusion

Taking in account these present available data, the mechanism of action of P-MAPA was clearly distinct in relation to BCG. These important findings are relevant concerning the treatment of patients with NMIBC presenting high risk of progression that are refractory or resistant to intravesical therapy with BCG.

## Results

### P-MAPA reverses the histopathological changes induced by MNU

**Fig. 2:**
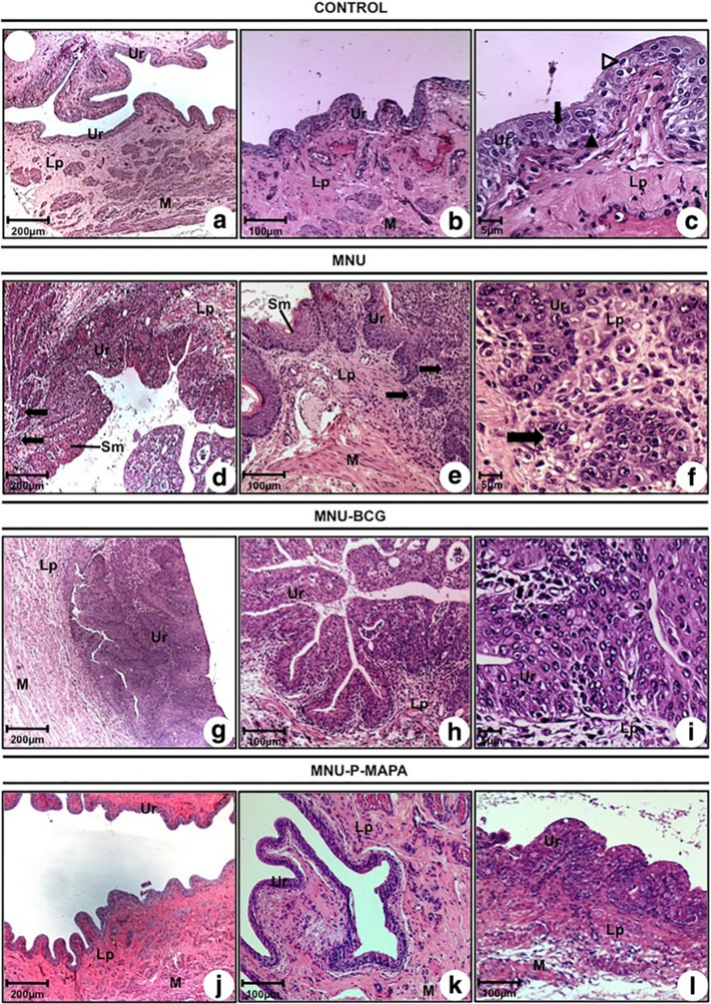
**a–l** Photomicrographs of the urinary bladder from CONTROL (**a, b, c**), MNU (**d, e, f**), MNU-BCG (**g, h, i**) and MNU-P-MAPA (**j, k, l**) groups. **a**, **b**, **c**, **j** and **k** Normal urothelium composed of 2–3 layers: a basal cell layer (**arrowhead**), an intermediate cell layer (**arrow**), and a superficial or apical layer composed of umbrella cells (**open arrowhead**). **d**, **e** and **f** pT1: neoplastic cells arranged in small groups (**arrows**) invading the lamina propria; keratinizing squamous metaplasia (**Sm**). **g**, **h** and **i** pTa characterized by fibrovascular stalk and frequent papillary branching with increased cellular size. **l** Papillary hyperplasia. **a**–**l** *Lp* lamina propria, *M* muscular layer, *Ur* urothelium

The urinary tract from the CONTROL group showed no microscopic changes (Fig. 2a, b and c; Additional file 1: Table S1). The normal urothelium was composed of three layers: a basal cell layer, an intermediate cell layer, and a superficial layer composed of umbrella cells (Fig. 2a, b, c).

In contrast, the urinary bladders from the MNU group showed histopathological changes such as tumor invading mucosa or submucosa of the bladder wall (pT1) (Fig. 2d, e and f), papillary carcinoma non-invasive (pTa) and flat carcinoma in situ (pTis) in 40, 40 and 20 % of the animals, respectively (Additional file 1: Table S1). The keratinizing squamous metaplasia was found in 60 % of the animals (Fig. 2d and e).

The most frequent histopathological changes in the urinary bladder from the MNU-BCG group were pTa (Fig. 2g, h and i; Additional file 1: Table S1) low-grade intraurothelial neoplasia and papillary hyperplasia in 40, 40 and 20 % of the animals, respectively (Additional file 1: Table S1).

The microscopic features of the urinary bladders from the MNU-P-MAPA group were similar to those found in the CONTROL group (Fig. 2j, k and l). Normal urothelium was found in 60 % of the animals (Fig. 2j and k; Additional file 1: Table S1). The histopathological changes in the MNU-P-MAPA group were flat hyperplasia (20 %) and papillary hyperplasia (20 %) (Fig. 2l; Additional file 1: Table S1).

Urinary calculi and macroscopic haematuria were only observed in the MNU and MNU-BCG groups; they were absent in the MNU-P-MAPA group.

### BCG activates MyD88-dependent pathway

**Fig. 3:**
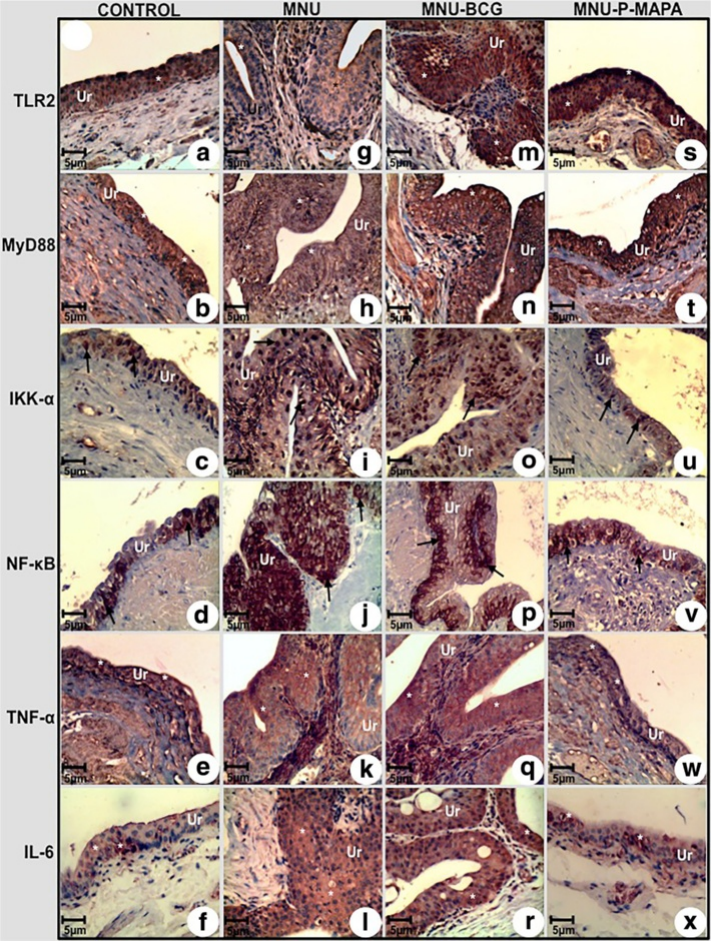
Immunolabelled antigen intensities of the urinary bladder from the CONTROL (**a, b, c, d, e, f**), MNU (**g, h, i, j, k, l**), MNU-BCG (**m, n, o, p, q, r**), and MNU-P-MAPA (**s, t, u, v, w, x**) groups. TLR2 immunoreactivities (**asterisks**) were moderate in the urothelium from the CONTROL (**a**) group, weak in the MNU (**g**) group and intense in the MNU-BCG (**m**) and MNU-P-MAPA (**s**) groups. MyD88 immunoreactivities (**asterisks**) were moderate in the urothelium from the CONTROL (**b**) group, weak in the MNU (**h**) group and intense in the MNU-BCG (**n**) and MNU-P-MAPA (**t**) groups. IKK-α immunoreactivities (**arrows**) were weak in the urothelium from the CONTROL (**c**) group, moderate in the MNU (**i**) group, intense in the MNU-BCG group (**o**) and weak in the MNU-P-MAPA (**u**) group. NF-kB immunoreactivities (**arrows**) were weak in the cytoplasm of the urothelial cells from the CONTROL (**d**) group, intense in the nucleus and cytoplasm of the urothelial cells from the MNU (**j**) group, moderate in the nucleus and cytoplasm of the urothelial cells from the MNU-BCG (**p**) group and weak in the cytoplasm of the urothelial cells from the MNU-P-MAPA (**v**) group. TNF-α immunoreactivities (**asterisks**) were weak in the urothelium from the CONTROL (**e**) group, intense in the MNU (**k**) and MNU-BCG (**q**) groups and weak in the MNU-P-MAPA (**w**) group. IL-6 immunoreactivities (**asterisks**) were weak in the urothelium from the CONTROL (**f**) group, intense in the MNU (**l**) and MNU-BCG (**r**) groups and weak in the MNU-P-MAPA (**x**) group. **a–x** *Ur* urothelium

**Fig. 4:**
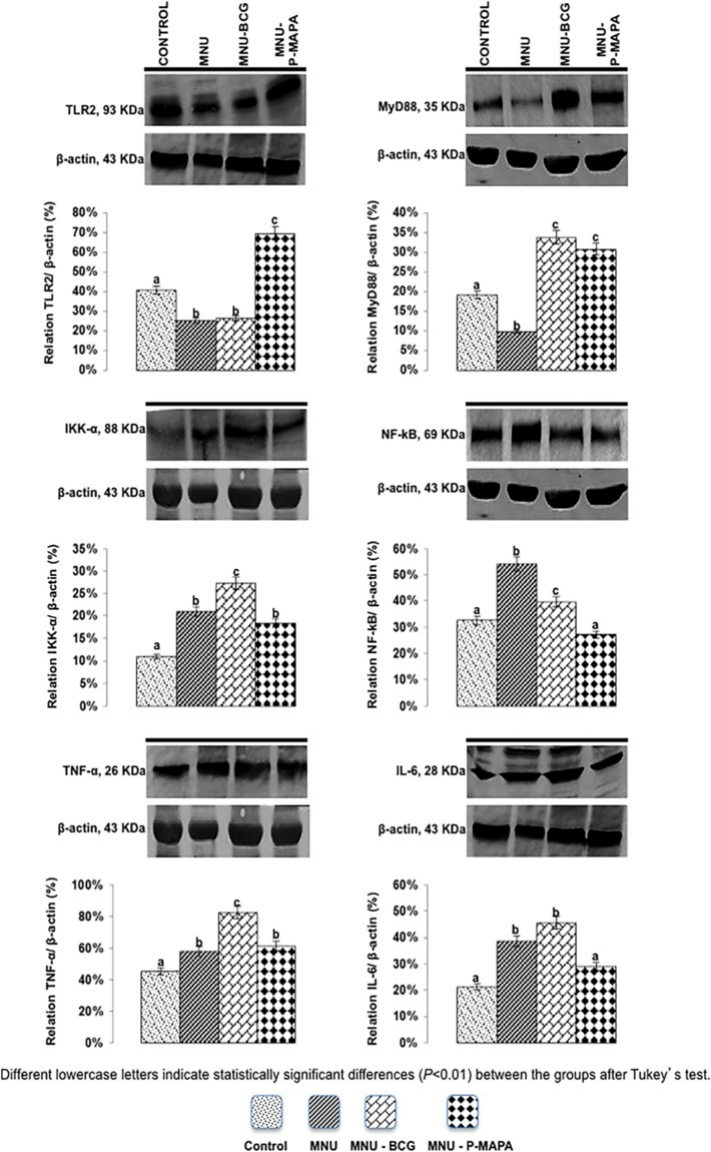
Representative Western Blotting and semiquantitative determination for TLR2, MyD88, IKK-α, NF-kB, TNF-α and IL-6 protein levels. Samples of urinary bladder were pooled from five animals per group for each repetition (duplicate) and used for semi-quantitative densitometry (IOD – Integrated Optical Density) analysis of the TLR2, MyD88, IKK-α, NF-kB, TNF-α and IL-6 levels following normalization to the β-actin. All data were expressed as the mean ± standard deviation. Different lowercase letters (**a, b, c, d**) indicate significant differences (*p* <0.01) between the groups after Tukey’s test

The highest TLR2 protein levels were found in the MNU-P-MAPA group as compared to the CONTROL, MNU-BCG and MNU groups, showing intense immunoreactivities in the urothelium (Figs. 3a, g, m, s and 4; Additional file 2: Table S2).

The highest MyD88 protein levels were found in the MNU-BCG and MNU-P-MAPA groups as compared to the other experimental groups. These groups showed intense immunoreactivities in the urothelium (Figs. 3b, h, n, t and 4; Additional file 2: Table S2). However, MyD88 levels were significantly higher in the CONTROL group than in the MNU group; these groups exhibited moderate and weak immunoreactivities, respectively (Figs. 3b, h, n, t and 4; Additional file 2: Table S2).

IKK-α protein levels were significantly higher in the MNU-BCG group in relation to the MNU, MNU-P-MAPA and CONTROL groups, which showed intense, moderate, weak and weak immunoreactivities in the urothelium, respectively (Figs. 3c, i, o, u and 4; Additional file 2: Table S2).

The highest NF-kB protein levels were found in the MNU group as compared to the MNU-BCG, CONTROL and MNU-P-MAPA groups (Fig. 4). The NF-kB immunoreactivities were weak in the cytoplasm of the urothelial cells from the CONTROL group, intense in both nucleus and cytoplasm of the urothelial cells from the MNU group, moderate in both nucleus and cytoplasm of the urothelial cells from the MNU-BCG group, and weak in the cytoplasm of the urothelial cells from the MNU-P-MAPA group (Figs. 3d, j, p and v; Additional file 2: Table S2).

TNF-α protein levels were significantly higher in the MNU-BCG group than in all other experimental groups, exhibiting intense immunoreactivities in the urothelium (Figs. 3e, k, q, w and 4; Additional file 2: Table S2). However, these levels were significantly higher in the MNU-P-MAPA and MNU groups in relation to the CONTROL group, which showed weak, intense and weak immunoreactivities, respectively (Fig. 3e, k, q, w and 4; Additional file 2: Table S2).

IL-6 protein levels were significantly higher in the MNU-BCG and MNU groups in relation to the MNU-P-MAPA and CONTROL groups. These groups displayed intense, intense, weak and weak immunoreactivities in the urothelium, respectively (Figs. 3f, l, r, x and 4; Additional file 2: Table S2).

### P-MAPA intravesical immunotherapy activates interferon signaling pathway and increases iNOS levels

**Fig. 5:**
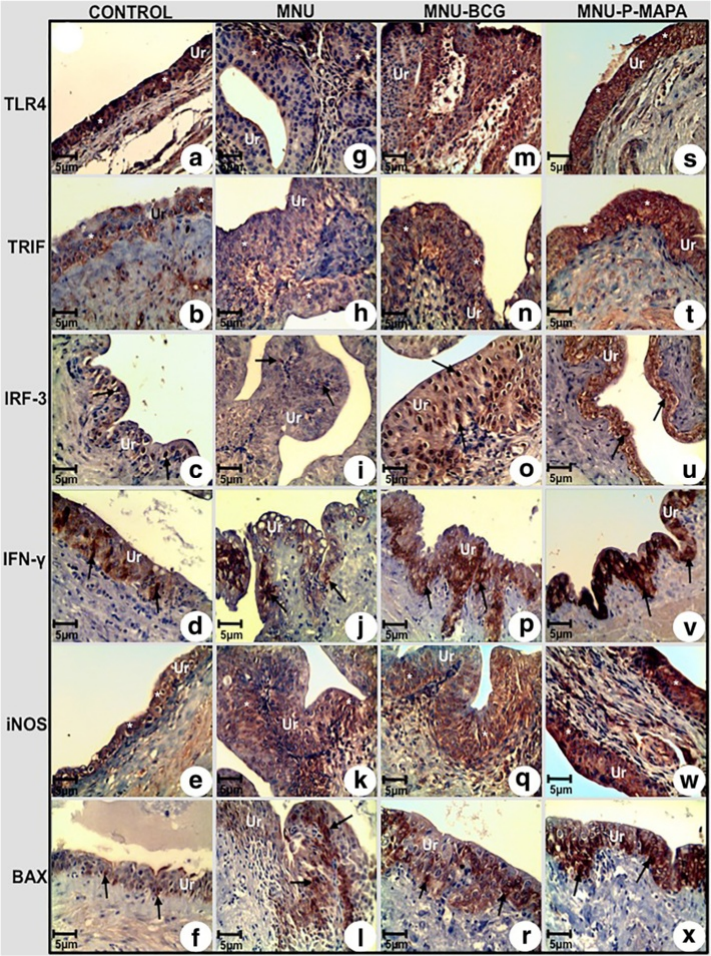
Immunolabelled antigen intensities of the urinary bladder from the CONTROL (**a, b, c, d, e, f**), MNU (**g, h, i, j, k, l**), MNU-BCG (**m, n, o, p, q, r**), and MNU-P-MAPA (**s, t, u, v, w, x**) groups. TLR4 immunoreactivities (**asterisks**) were moderate in the urothelium from the CONTROL group (**a**), weak in the MNU group (**g**) and intense in the MNU-BCG (**m**) and MNU-P-MAPA (**s**) groups. TRIF immunoreactivities (**asterisks**) were weak in the urothelium from the CONTROL (**b**) and MNU (**h**) groups, moderate in the MNU-BCG (**n**) group and intense in the MNU-P-MAPA (**t**) group. IRF-3 immunoreactivities (**arrows**) were weak in the urothelium from the CONTROL (**c**) and MNU (**i**) groups, moderate in the MNU-BCG (**o**) group and intense in the MNU-P-MAPA (**u**) group. IFN-γ immunoreactivities (**arrows**) were weak in the urothelium from the CONTROL (**d**) and MNU (**j**) groups, moderate in the MNU-BCG (**p**) group and intense in the MNU-P-MAPA (**v**) group. iNOS immunoreactivities (**asterisks**) were weak in the urothelium from the CONTROL (**e**) and MNU (**k**) groups, moderate in the MNU-BCG (**q**) group and intense in the MNU-P-MAPA (**w**) group. BAX immunoreactivities (**asterisks**) were weak in the urothelium from the CONTROL (**f**) group, moderate in the MNU (**l**) and MNU-BCG (**r**) groups and intense in the MNU-P-MAPA (**x**) group. **a–x** *Ur* urothelium

**Fig. 6:**
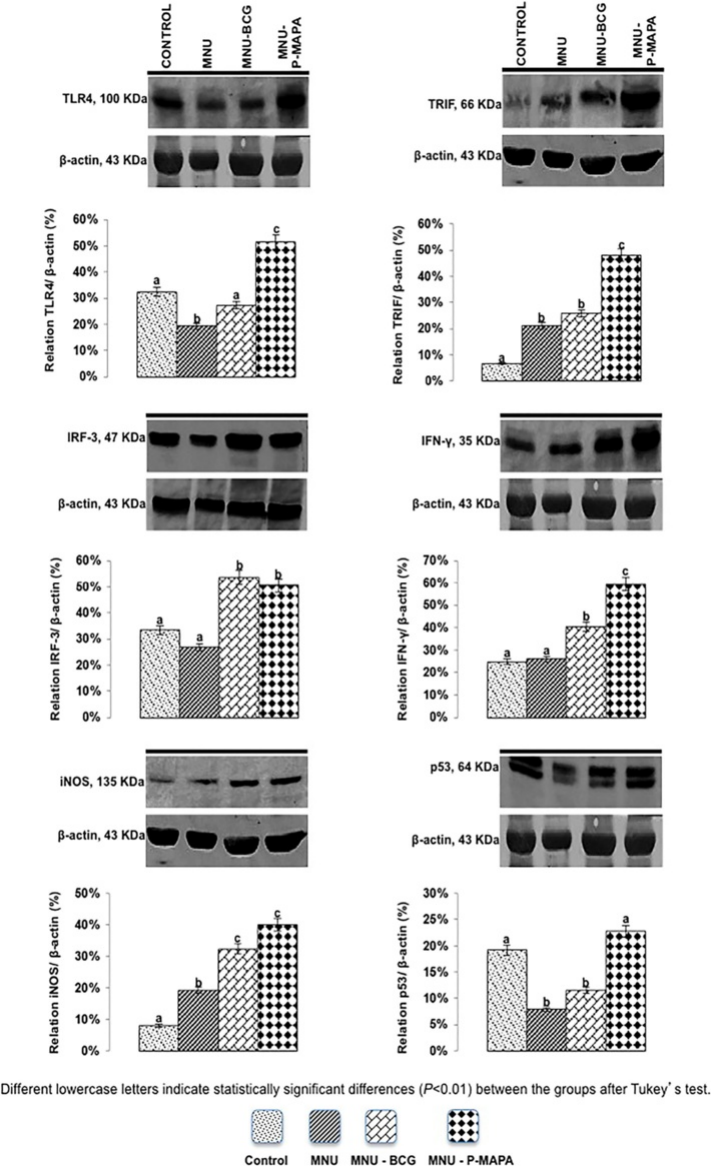
Representative Western Blotting and semiquantitative determination for TLR4, TRIF, IRF-3, IFN-γ, iNOS, and p53 protein levels. Samples of urinary bladder were pooled from five animals per group for each repetition (duplicate) and used for semi-quantitative densitometry (IOD – Integrated Optical Density) analysis of the TLR4, TRIF, IRF-3, IFN-γ, iNOS, and p53 levels following normalization to the β-actin. All data were expressed as the mean ± standard deviation. Different lowercase letters (**a, b, c, d**) indicate significant differences (*p* <0.01) between the groups after Tukey’s test

TLR4 protein levels were significantly higher in the MNU-P-MAPA group in relation to the other experimental groups. This group exhibited intense immunoreactivities in the urothelium (Figs. 5a, g, m, s and 6; Additional file 2: Table S2). However, these levels were significantly higher in the CONTROL and MNU-BCG groups than in the MNU group. The three latter groups showed moderate, intense and weak immunoreactivities, respectively (Figs. 5a, g, m, s and 6; Additional file 2: Table S2).

TRIF protein levels were significantly higher in the MNU-P-MAPA group in relation to the other experimental groups, which showed intense immunoreactivities in the urothelium (Figs. 4b, h, n, t and 6; Additional file 2: Table S2). However, TRIF levels were higher in the MNU-BCG and MNU groups than in the CONTROL group. The three latter groups exhibited moderate, weak and weak immunoreactivities respectively (Figs. 5b, h, n, t and 6; Additional file 2: Table S2).

Protein levels for IRF-3 were significantly higher in the MNU-BCG and MNU-P-MAPA groups in relation to the CONTROL and MNU groups. These groups showed moderate, intense, weak and weak immunoreactivities in the urothelium, respectively (Figs. 5c, i, o, u and 6; Additional file 2: Table S2).

The highest IFN-γ protein levels were found in the MNU-P-MAPA group compared to the MNU-BCG, MNU and CONTROL groups. These groups exhibited intense, moderate, weak and weak immunoreactivities in the urothelium, respectively (Figs. 5d, j, p, v and 6; Additional file 2: Table S2).

iNOS protein levels were significantly higher in the MNU-P-MAPA and MNU-BCG groups than in the MNU and CONTROL groups. These groups showed intense, moderate, weak and weak immunoreactivities in the urothelium, respectively (Figs. 5e, k, q, w and 6; Additional file 2: Table S2).

**Fig. 7:**
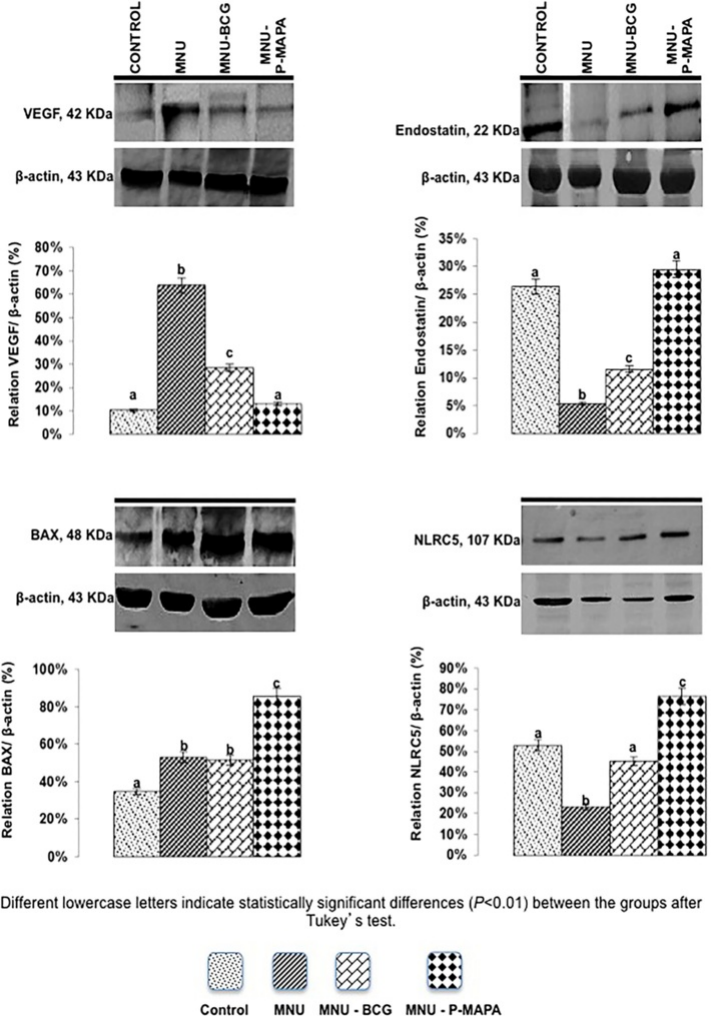
Representative Western Blotting and semiquantitative determination for VEGF, Endostatin, BAX and NLRC5 protein levels. Samples of urinary bladder were pooled from five animals per group for each repetition (duplicate) and used for semi-quantitative densitometry (IOD – Integrated Optical Density) analysis of the VEGF, Endostatin, BAX and NLRC5 levels following normalization to the β-actin. All data were expressed as the mean ± standard deviation. Different lowercase letters (**a, b, c, d**) indicate significant differences (*p* <0.01) between the groups after Tukey’s test

NLRC5 protein levels were significantly higher in the MNU-P-MAPA group in relation to the other experimental groups (Fig. 7). Furthermore, these levels were significantly higher in the CONTROL and MNU-BCG groups than in the MNU group (Fig. 7).

### P-MAPA immunotherapy increases wild-type p53 protein levels, decreases proliferation and increases apoptosis

p53 protein levels were significantly higher in the MNU-P-MAPA and CONTROL groups in relation to the other experimental groups (Fig. 6). Furthermore, these levels were significantly higher in the MNU-BCG group in comparison to the MNU group (Fig. 6).

**Fig. 8:**
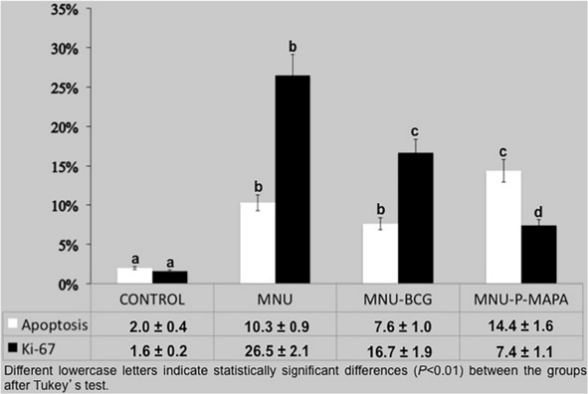
Percentage of Proliferative (Ki-67) and Apoptotic Indexes

The apoptotic index revealed different kinetics for cell death for each treatment (Additional file 3: Figures S1a, S1c, S1e, S1g; Fig. 8). This index was significantly higher in the animals from the MNU-P-MAPA group in relation to the other experimental groups. The MNU and MNU-BCG groups, in turn, showed significantly higher average values of the apoptotic index than the CONTROL group (Additional file 3: Figures S1a, S1c, S1e, S1g; Fig. 8). BAX protein levels were significantly higher in the MNU-P-MAPA group compared to the MNU, MNU-BCG and CONTROL groups. The groups exhibited intense, moderate, moderate and weak immunoreactivities in the urothelium, respectively (Figs. 3f, l, r, x and 6; Additional file 2: Table S2).

Proliferative activity was significantly increased in animals from the MNU group in relation to the other experimental groups (Additional file 3: Figures S1b, S1d, S1f, S1h; Fig. 8). The MNU-P-MAPA group displayed significantly lower average values of proliferative index than the MNU-BCG group, although these values were significantly higher than those found in the CONTROL group (Additional file 3: Figures S1b, S1d, S1f, S1h; Fig. 8).

**Fig. 9:**
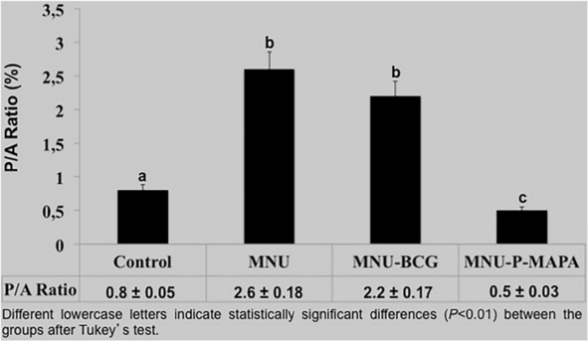
Proliferation/Apoptotic Ratio (P/A)

Furthermore, the proliferation/apoptotic ratio (P/A) was significantly higher in the MNU and MNU-BCG groups when compared to CONTROL group (Fig. 9). However, the P/A ratio in the MNU-P-MAPA was significantly lower in relation to the other experimental groups, indicating predominance of the apoptotic process (Fig. 9).

### P-MAPA intravesical immunotherapy suppresses angiogenesis

VEGF protein levels were significantly higher in the MNU group in relation to the other experimental groups (Fig. 7). Furthermore, these levels were significantly higher in the MNU-BCG group compared to the MNU-P-MAPA and CONTROL groups (Fig. 7).

Endostatin protein levels were significantly higher in the MNU-P-MAPA and CONTROL groups when compared to the MNU-BCG and MNU groups (Fig. 7).

## Discussion

Although the use of TURBT with adjuvant chemo and immunotherapy represents a clear advance in the treatment of NMIBC, the management of this disease, mainly for high grade tumors remains a challenge, because the high rates of recurrence and progression to muscle invasive and/or metastatic stages. Following episodes of high grade NMIBC recurrence after BCG therapy, several conventional chemotherapy agents have been used including gemcitabine, mitomycin, gemcitabine plus mitomycin, docetaxel and valrubicin. In addition, immunotherapy (Interferon-alpha or Interferon alpha-plus BCG) has also been used [31]. *Mycobacterium phlei* cell wall-nucleic acid complex (MCNA) has been proposed for intravesical treatment of NMIBC at high risk of recurrence or progression in patients who failed prior BCG immunotherapy (e.g., in patients who are BCG-refractory or BCG relapsing) and are not candidates for or refuse cystectomy [32]. However, none of these drugs had been shown superiority over BCG and remains considered investigational [14]. In the specific case of BCG-refractory CIS, Valrubicin, a semi-syntetic analog of doxorubicin, the only FDA-approved drug for treatment of such condition, shows effectivity in less of 10 % of treated patients at 2 years and none with coincident stage T1 disease [33].

The surgical option for such cases, partial or total cystectomy, is often associated with significant morbidity and mortality. Furthermore, for some patients, cystectomy is not an available option due to the presence of concomitant comorbidities. Consequently novel therapies are highly needed for treatment of high grade NMIBC, to prevent disease progression and to allow bladder preservation and ensure life quality for patients and finally, to provide an option for those that are ineligible for cystectomy.

The P-MAPA Biological Response Modifier, which shows novel therapeutic properties compared to standard treatments, appears a valuable candidate drug in the treatment of NMIBC. In our previous studies we have shown several beneficial properties of P-MAPA [11, 12]. Here, using the NMU animal models for the study of NMIBC, we clearly show that P-MAPA treatment enables better histopathological recovery from the cancer state than no treatment (MNU group) or BCG treatment (MNU-BCG group).

Agonists of TLRs are the subject of intensive research and development for the treatment of cancer, including bladder cancer [11, 12, 33]. TLRs, which are expressed in immune as well as in some epithelial cells, play an important role in activating both innate and adaptive immune responses [33] and [33, 34]. Bladder tumors, especially non-muscle-invasive, show decreased TLRs expression [35, 36]. TLR-mediated BCG immunotherapy for NMIBCs suggests alternative TLR-based immunotherapies might also be successful strategies for this type of cancer. The BCG antitumor effects seem to be related to local immunological mechanisms since after BCG instillation, a transient increase in several cytokines and the presence of activated immunocompetent leukocytes were found in the urine within 24 h [37]. Local lymphocytic infiltration and cytokine production were found in the bladder wall of most patients receiving intravesical BCG and was demonstrated that this local response was highly complex [37–39]. TNF-related-apoptosis-inducing ligand (TRAIL) is released from polymorph nuclear neutrophils (PMNs) via stimulation of TLR2 by BCG [33]. Secretion of interleukin-8, a strong chemoattractant for monocytes and T-cells, is also induced from PMNs by BCG infection via MyD88-dependent TLR2 and TLR4 activation [33, 40] whereas BCG activation of TLR2 and TLR4 induces TNF-α secretion from dendritic cells (DCs) [33, 41, 42].

TNF signaling pathway may induce carcinogenesis by up-regulating NF-kB leading to the up-regulation of other proteins that cause cell proliferation and morphogenesis [40]. Using TNF knockout mice the development of skin carcinomas by chemical carcinogen DMBA (7.12-dimethylbanz[a]-antracene) and tumor promoter TPA (12-0-tetradecanoyl-phorbol-13-acetate) decreased compared to wild type mice [43, 44]. Using pentoxifylline, which was shown to inhibit TNF and IL-1a gene expression, the growth of DMBA/TPA induced papillomas were inhibited [45]. These results suggest a chemical tumor promoter can induce the secretion of TNF-α from different cells types and TNF can act as an endogenous tumor promoter in vivo [46]. TNF-α was identified as the major host-produced factor that enhances the growth of metastases in the lung cancer animal model, in part through activation of NF-kB in the tumor cells [47].

We have demonstrated here that BCG increased TLR2 and TLR4 protein levels in NMIBC model, which corroborated with our previous study [11, 12]. These induces MyD88-dependent pathway as shown by increased MyD88, IKKα and NF-kB protein levels. The induction of MyD88-dependent pathway or canonical pathway increases inflammatory cytokines (IL-6 and TNF-α) protein levels. Accordingly, activation of immune system by BCG treatment, via MyD88-dependent pathway, (Additional file 4: Figure S2a), was essential for histopathological recovery from the cancer state.

TLR4 activation of host macrophages resulted in the production of several different inflammatory cytokines that influenced tumor growth. However, TLR4 signaling also induces cytokines (IFN) that have antitumor effects by induction of TRAIL, a potent inducer of tumor cell death [47]. Shankaran et al. [48] showed the tumorsuppressor function of the immune system to be critically depend on the actions of IFN-γ, which, at least in part, are driven to regule tumor-cell immunogenicity. IFN-γ stimulates several antiproliferative and tumoricidal biochemical pathways in macrophages and in tumor cell lines, as well as has a profound impact on solid tumors growth and metastasis and seemingly plays an early role in protection from metastasis [49–55]. IFN-γ produced by IL-12-activated tumor-infiltrating CD8+T cells directly induced apoptosis of mouse hepatocellular carcinoma cells [52, 53]. The NLRCs, a class of intracellular receptors that respond to pathogen or cellular stress, has recently been identified as a critical regulator of immune responses [56, 57]. While NLRC5 is constitutively and widely expressed, its levels can be dramatically induced by interferons during pathogen infections. Both in vitro and in vivo studies have demonstrated NLRC5 is a specific and master regulator of major histocompatibility complex (MHC) class I genes as well as related genes involved in MHC class I antigen presentation [56, 57].

In this study, we demonstrated TLR2 and TLR4 protein levels were significantly higher in the P-MAPA group in relation to the BCG group in the NMIBC animal model. Also, P-MAPA treatment led to increased TRIF and IRF-3 protein levels, indicating an activation of MyD88-independent pathway (Additional file 4: Figure S2b). The induction of MyD88-independent pathway (non-canonical pathway or TRIF-dependent pathway) by P-MAPA led to increased IFN-γ and iNOS (macrophages type 1 – M1) protein levels. In contrast to BCG treatment, P-MAPA immunotherapy led to distinct activation of innate immune system TLRs 2 and 4-mediated, resulting in increased interferons signaling pathway (Additional file 4: Figure S2b), which was more effective in the NMIBC treatment. Also as result of interferon signaling pathway (IFN-γ and IRF-3) induction by P-MAPA, the proliferation/apoptotic ratio was significantly lower in animals treated with P-MAPA, indicating predominance of the apoptotic process. Accordingly, P-MAPA immunotherapy increased NOD like receptor 5 (NLRC5) protein levels, which were fundamental to induction of interferon signaling pathway (Additional file 4: Figure S2b). Thus, the activation of interferon signaling pathway was more effective in the induction of immunogenic cell death in relation to inflammatory cytokines signaling pathway.

The IFN-γ produced by tumor-infiltrating T cells might play two distinct roles in antitumor activity: activation of antitumor T cells and direct tumoricidal activity by generating inducible nitric oxide synthetase (iNOS) [48, 58]. NO is considered one of the main factors responsible for the macrophage cytotoxic activity against tumor cells [50, 59]. Previous data showing increased NO concentrations in the urinary bladder from patients treated with BCG [59–61], suggests NO as a critical factor in the BCG mediated antitumor effect [56]. NO can stimulate cell growth and cell differentiation when present at low concentrations, whereas high concentrations often result in cytotoxic effects [59]. Tate et al. [50] demonstrated iNOS induction within the renal carcinoma cells (CL-2 and CL-19) in response to IFN-γ caused a robust and sustained accumulation of endogenous NO that resulted in an 80–85 % growth inhibition of CL-2 and CL-19 cell lines. Patients with bladder cancer who had received BCG treatment, iNOS-like immunoreactivity was found in the urothelial cells but also in macrophages in the submucosa [56]. Koskela et al. [59] verified that endogenously formed NO was significantly increased in the BCG treated patients and they had a ten-fold increase in mRNA expression for iNOS compared to healthy controls. In culture supernatant from macrophages stimulated by P-MAPA in both healthy and visceral leishmaniasis, infected dogs NO production was increased [62]. Thus, it can be concluded interferon signaling pathway activation induced by P-MAPA led to increase of iNOS protein levels in the NMIBC animal model, resulting in increased apoptosis process and histopathological recovery (Additional file 4: Figure S2b).

Furthermore, cell death may depend on NO-stimulated signaling pathways leading to gene expression, involving the tumor suppressor *p53* [63–65]. Activation of *p53* by NO has been observed in many cell types [66, 67]. NO-induced *p53* contributes to various cell type-specific biological effects of NO, such as induction of apoptosis, inhibition of proliferation and tumor suppression [66–68]. Besides that, p53 controls a remarkable number of physiologic functions, including energy metabolism, differentiation, and reactive oxygen species production and is stabilized and activated in response to diverse stresses signals, such as DNA damage, hypoxia, oncogene activation, drugs, nucleotide depletion [64]. Cells possessing a fully functional p53 pathway can either arrest and repair damages caused by these untimely stresses or undergo p53-dependent apoptosis. BAX is considered an important target gene required for p53-dependent apoptosis [64]. Induction of p53 by NO is preceded by a rapid decrease in Mdm2 protein, which may enable to elevate p53 levels early after exposure to NO [67]. Wang et al. [67] showed NO promoted p53 nuclear retention and inhibited Mdm2-mediated p53 nuclear export, indicating this effect to be mediated by ATM-dependent phosphorylation of p53 on Serine 15. Also, In conclusion, these findings imply that, through augmenting p53 nuclear retention NO can sensitize tumor cells to p53-dependent apoptosis.

Several studies suggest antiangiogenic therapy is sensitive to p53 status in tumors, implicating a role for p53 in the regulation of angiogenesis [18, 19, 69]. A connection between p53 and tumor angiogenesis was revealed in 1994 when Dameron et al. [69] proposed suppression of angiogenesis by thrombospondin-1 could represent a new mechanism for tumor suppression by p53. Other evidence emerged that wild-type p53 could prevent incipient tumors from becoming angiogenic [70]. Teodoro et al. [19] demonstrated p53-tumor suppression was mediated in part by at least two potent angiogenesis inhibitors, endostatin and tumstatin. In addition, these authors showed ectopic expression of α (II) collagen prolyl-4-hydroxylase in human tumor cells implanted into immunodeficient mice resulted in “near-complete” tumor suppression compared with mice implanted with tumor cells that did not express α (II) collagen prolyl-4-hydroxylase, and associated this results with suppression of tumor angiogenesis by endostatin or tumstatin. Thus, this study demonstrated an important antitumor effect of P-MAPA immunotherapy, based on increase of endostatin protein levels and decrease of VEGF protein levels in the NMIBC animal model. Therefore, interferon signaling pathway induction and increased wild-type p53 protein levels by P-MAPA led to important antitumor effects, not only suppressing abnormal cell proliferation, but also by preventing continuous expansion of tumor mass through suppression of angiogenesis.

## Conclusions

Taking in account these present available data, the mechanism of action of P-MAPA was clearly distinct in relation to BCG. These important findings are relevant concerning the treatment of patients with NMIBC presenting high risk of progression that are refractory or resistant to intravesical therapy with BCG. 

## Abbreviations

BAX, bcl-2-like protein 4; BC, bladder cancer; BCG, Bacillus Calmette-Guerin; BSA, bovine serum albumin; HRP, horseradish peroxidase; IFN-γ, interferon-gamma; IL, interleukin; IL-6, interleukin 6; iNOS, inducible nitric oxide synthetase; IRF-3, interferon regulatory factor 3; MNU, n-methyl-n-nitrosourea; MyD88, myeloid differentiation primary response 88; NF-kB, nuclear factor-kB; NK, natural killer cell; NLRC5, NOD like receptor 5; NMIBC, non-muscle invasive bladder cancer; NO, nitric oxide; P-MAPA, protein aggregate magnesium-ammonium phospholinoleate-palmitoleate anhydride; pT1, tumor confined to the mucosa and submucosa of the bladder; pTa, papillary tumor; pTis, carcinoma in situ; TLR, toll-like receptor; TNF-α, tumor necrosis factor α; TRAF2, TNF receptor-associated factor 2; TRIF, TIR-domain-containing adapter-inducing interferon-β

## Additional files

Additional file 1: Table S1. Percentage of histopathological changes of the urinary bladder of rats from CONTROL, MNU, MNU-BCG and MNU-P-MAPA groups. (DOCX 61 kb) Additional file 2: Table S2. Semiquantitative analysis of immunolabelled antigens of the urinary bladder of rats in the different experimental groups. (DOCX 165 kb) Additional file 3: Figures S1a–S1h. Immunolabelled Ki-67 intensities and detection of apoptosis in the urinary bladder from the CONTROL (a, b), MNU (c, d), MNU-BCG (e, f), and MNU-P-MAPA (g, h) groups. (a), (c), (e) and (g) DNA fragmentation (**arrows**) in the urothelium. (b), (d), (f) and (h) Ki-67 immunoreactivities (**arrows**) in the urothelium. a–h: *Ur* urothelium. (JPG 1261 kb) Additional file 4: Figures S2a–S2b. (a) Schematic representation of the mechanism of action of BCG involving TLRs signaling pathway. (b) Hypothetical mechanism of P-MAPA immunotherapy (Developed by Wagner José Fávaro and Farmabrasilis). (JPG 1048 kb) Additional file 5: Availability of Data and Materials (DOCX 25 kb) Additional file 6: Ethics Approval (JPG 1348 kb)

## References

[1] American Cancer Society. Bladder Cancer Statistics. 2015. http://www.cancer.org/cancer/bladdercancer/detailedguide/bladder-cancer-key-statistics. Accessed at 10 Dec 2015.

[2] Zhang N, Li D, Shao J, Wang X (2015). Animal models for bladder cancer: the model establishment and evaluation. Oncol Lett.

[3] Shimada K, Fujii T, Anai S, Fujimoto K, Konishi N (2011). ROS generation via NOX4 and its utility in the cytological diagnosis of urothelial carcinoma of the urinary bladder. BMC Urol.

[4] Epstein JI, Amin MB, Reuter VR, Mostofi FK (1998). The World Health Organization/International Society of Urological Pathology consensus classification of urothelial (transitional cell) neoplasms of the urinary bladder. Bladder Consensus Conference Committee. Am J Surg Pathol.

[5] Askeland EJ, Newton MR, O’Donnell MA, Luo Y (2012). Bladder cancer immunotherapy: BCG and Beyond. Adv Urol.

[6] Böhle A, Brandau S (2003). Immune mechanisms in bacillus Calmette Guerin Immunotherapy for superficial bladder cancer. J Urol.

[7] DiPaola RS, Lattime EC (2007). Bacillus Calmette-Guerin mechanism of action: role of immunity, apoptosis, necrosis and autophagy. J Urol.

[8] Berry DL, Blumenstein BA, Magyary DL, Lamm DL, Crawford ED (1996). Local toxicity patterns associated with intravesical bacillus Calmette-Guerin: a Southwest Oncology Group study. Int J Urol.

[9] Herr HW, Milan TN, Dalbagni G (2015). BCG-refractory vs. BCG-relapsing non-muscle-invasive bladder cancer: a prospective cohort outcomes study. Urol Oncol.

[10] Killeen SD, Wang JH, Andrews EJ, Redmond HP (2006). Exploitation of the Toll like receptor system in cancer: a doubled-edged sword?. Br J Cancer.

[11] Fávaro WJ, Nunes OS, Seiva FR, Nunes IS, Woolhiser LK, Duran N (2012). Effects of P-MAPA immunomodulator on Toll-like receptors and p53: potential therapeutic strategies for infectious diseases and cancer. Infect Agent Cancer.

[12] Garcia PV, Apolinário LM, Böckelmann PK, da Silva NI, Duran N, Fávaro WJ (2015). Alterations in ubiquitin ligase Siah-2 and its corepressor N-CoR after P-MAPA immunotherapy and anti-androgen therapy: new therapeutic opportunities for non-muscle invasive bladder cancer. Int J Clin Exp Pathol.

[13] Akira S, Takeda K (2004). Toll-like receptor signalling. Nat Rev Immunol.

[14] Takeda K, Akira S (2004). TLR signaling pathways. Semin Immunol.

[15] Zhao S, Zhang Y, Zhang Q, Wang F, Zhang D (2014). Toll-like receptors and prostate cancer. Front Immunol.

[16] Menendez D, Shatz M, Azzam K (2011). The Toll-like receptor gene family is integrated into human DNA damage and p53 networks. Plos Genet.

[17] Shariat SF, Lotan Y, Karakiewicz PI, Ashfaq R, Isbarn H, Fradet Y (2009). p53 predictive value for pT1-2 N0 disease at radical cystectomy. J Urol.

[18] Folkman J (2006). Antiangiogenesis in cancer therapy--endostatin and its mechanisms of action. Exp Cell Res.

[19] Teodoro JG, Parker AE, Zhu X, Green MR (2006). p53-mediated inhibition of angiogenesis through up-regulation of a collagen prolyl hydroxylase. Science.

[20] Verdegem D, Moens S, Stapor P, Carmeliet P (2014). Endothelial cell metabolism: parallels and divergences with cancer cell metabolism. Cancer Metab.

[21] Waltenberger J (2009). VEGF resistance as a molecular basis to explain the angiogenesis paradox in diabetes mellitus. Biochem Soc Trans.

[22] Zhu W, He S, Li Y, Qiu P, Shu M, Ou Y (2010). Anti-angiogenic activity of triptolide in anaplastic thyroid carcinoma is mediated by targeting vascular endothelial and tumor cells. Vascul Pharmacol.

[23] Abdollahi A, Lipson KE, Sckell A, Zieher H, Klenke F, Poerschke D (2003). Combined therapy with direct and indirect angiogenesis inhibition results in enhanced antiangiogenic and antitumor effects. Cancer Res.

[24] O’Reilly MS, Bohem T, Shing Y, Fukai N, Vasios G, Lane WS (1997). Endostatin: an endogenous inhibitor of angiogenesis and tumor growth. Cell.

[25] Nunes OS (1985). Desenvolvimento de um novo antibiótico. Reunião Anual da Sociedade Brasileira para o Progresso da Ciência, 37, 1985.

[26] Duran N, Nunes OS (1990). Characterization of an aggregated polymer from Penicilium sp. (PB 73 STRAIN). Braz J Med Biol Res.

[27] Duran N (1993). SB-73 immunostimulant. Drugs Future.

[28] Duran N (1997). SB-73/MAPA. Drugs Future.

[29] Farmabrasilis. The Farmabrasilis register. http://www.farmabrasilis.org (1987). Accessed 01 Dec 2015.

[30] Melo LM, Perosso J, Almeida BF, Silva KL, Somenzani MA, de Lima VM (2014). Effects of P-MAPA immunomodulator on Toll-like receptor 2, ROS, nitric oxide, MAPKp38 and IKK in PBMC and macrophages from dogs with visceral leishmaniasis. Int Immunopharmacol.

[31] Lightfoot AJ, Rosevear HM, O’Donnell MA (2011). Recognition and treatment of BCG failure in bladder cancer. Sci World J.

[32] Morales A, Herr H, Steinberg G, Given R, Cohen Z, Amrhein J, Kamat AM (2015). Efficacy and safety of MCNA in patients with nonmuscle invasive bladder cancer at high risk for recurrence and progression after failed treatment with bacillus Calmette-Guérin. J Urol.

[33] Steinberg GD, Smith ND, Ryder K, Strangman NM, Slater SJ (2011). Factors affecting valrubicin response in patients with bacillus Calmette-Guérin-refractory bladder carcinoma in situ. Postgrad Med.

[34] LaRue H, Ayari C, Bergeron A, Fradet Y (2013). Toll-like receptors in urothelial cells--targets for cancer immunotherapy. Nat Rev Urol.

[35] Ayari C, Bergeron A, LaRue H, Ménard C, Fradet Y (2011). Toll-like receptors in normal and malignant human bladders. J Urol.

[36] Stopiglia RM, Matheus W, Garcia PV, Billis A, Castilho MA, De Jesus VHF, Ferreira U, Fávaro WJ (2015). Molecular assessment of non-muscle invasive and muscle invasive bladder tumors: mapping of putative urothelial stem cells and Toll-Like Receptors (TLR) signaling. J Cancer Ther.

[37] Yu JS, Peacock JW, Jacobs WR, Frothingham R, Letvin NL, Liao HX, Haynes BF (2007). Recombinant Mycobacterium bovis bacillus Calmette-Guerin elicits human immunodeficiency virus type 1 envelope-specific T lymphocytes at mucosal sites. Clin Vaccine Immunol.

[38] Boccafoschi C, Montefiore F, Pavesi M, Pastormerlo M, Betta PG (1995). Late effects of intravesical bacillus Calmette-Guérin immunotherapy on bladder mucosa infiltrating lymphocytes: an immunohistochemical study. Eur Urol.

[39] Sander B, Damm O, Gustafsson B, Andersson U, Håkansson L (1996). Localization of IL-1, IL-2, IL-4, IL-8 and TNF in superficial bladder tumors treated with intravesical bacillus Calmette-Guerin. J Urol.

[40] Godaly G, Young DB (2005). Mycobacterium bovis bacille Calmette Guerin infection of human neutrophils induces CXCL8 secretion by MyD88-dependent TLR2 and TLR4 activation. Cell Microbiol.

[41] Tsuji S, Matsumoto M, Takeuchi O, Akira S, Azuma I, Hayashi A, Toyoshima K, Seya T (2000). Maturation of human dendritic cells by cell wall skeleton of Mycobacterium bovis bacillus Calmette-Guérin: involvement of toll-like receptors. Infect Immun.

[42] Simons MP, O’Donnell MA, Griffith TS (2008). Role of neutrophils in BCG immunotherapy for bladder cancer. Urol Oncol.

[43] Waterston AM, Salway F, Andreakos E, Butler DM, Feldmann M, Coombes RC (2004). TNF autovaccination induces self anti-TNF antibodies and inhibits metastasis in a murine melanoma model. Br J Cancer.

[44] Suganuma M, Okabe S, Marino MW, Sakai A, Sueoka E, Fujiki H (1999). Essential role of tumor necrosis factor alpha (TNF-alpha) in tumor promotion as revealed by TNF-alpha-deficient mice. Cancer Res.

[45] Robertson FM, Ross MS, Tober KL, Long BW, Oberyszyn TM (1996). Inhibition of pro-inflammatory cytokine gene expression and papilloma growth during murine multistage carcinogenesis by pentoxifylline. Carcinogenesis.

[46] Komori A, Yatsunami J, Suganuma M, Okabe S, Abe S, Sakai A (1993). Tumor necrosis factor acts as a tumor promoter in BALB/3T3 cell transformation. Cancer Res.

[47] Luo JL, Maeda S, Hsu LC, Yagita H, Karin M (2004). Inhibition of NF-kappaB in cancer cells converts inflammation- induced tumor growth mediated by TNFalpha to TRAIL-mediated tumor regression. Cancer Cell.

[48] Shankaran V, Ikeda H, Bruce AT, White JM, Swanson PE, Old LJ (2001). IFNgamma and lymphocytes prevent primary tumour development and shape tumour immunogenicity. Nature.

[49] Alshaker HA, Matalka KZ (2011). IFN-γ, IL-17 and TGF-β involvement in shaping the tumor microenvironment: the significance of modulating such cytokines in treating malignant solid tumors. Cancer Cell Int.

[50] Tate DJ, Patterson JR, Velasco-Gonzalez C, Carroll EN, Trinh J, Edwards D (2012). Interferon-gamma-induced nitric oxide inhibits the proliferation of murine renal cell carcinoma cells. Int J Biol Sci.

[51] Li Z, Pradera F, Kammertoens T, Li B, Liu S, Qin Z (2007). Cross-talk between T cells and innate immune cells is crucial for IFN-gamma-dependent tumor rejection. J Immunol.

[52] Komita H, Homma S, Saotome H, Zeniya M, Ohno T, Toda G (2006). Interferon-gamma produced by interleukin-12-activated tumor infiltrating CD8+T cells directly induces apoptosis of mouse hepatocellular carcinoma. J Hepatol.

[53] Martini M, Testi MG, Pasetto M, Picchio MC, Innamorati G, Mazzocco M (2010). IFN-gamma-mediated upmodulation of MHC class I expression activates tumor-specific immune response in a mouse model of prostate cancer. Vaccine.

[54] Street SE, Cretney E, Smyth MJ (2001). Perforin and interferon-gamma activities independently control tumor initiation, growth, and metastasis. Blood.

[55] duPre’ SA, Redelman D, Hunter KW (2008). Microenvironment of the murine mammary carcinoma 4T1: endogenous IFN-gamma affects tumor phenotype, growth, and metastasis. Exp Mol Pathol.

[56] Meissner N, Swain S, McInnerney K, Han S, Harmsen AG (2010). Type-I IFN signaling suppresses an excessive IFN-gamma response and thus prevents lung damage and chronic inflammation during Pneumocystis (PC) clearance in CD4 T cell-competent mice. Am J Pathol.

[57] Yao Y, Qian Y (2013). Expression regulation and function of NLRC5. Protein Cell.

[58] Beatty GL, Paterson Y (2001). Regulation of tumor growth by IFN-gamma in cancer immunotherapy. Immunol Res.

[59] Koskela LR, Poljakovic M, Ehrén I, Wiklund NP, de Verdier PJ (2012). Localization and expression of inducible nitric oxide synthase in patients after BCG treatment for bladder cancer. Nitric Oxide.

[60] Hosseini A, Koskela LR, Ehrén I, Aguilar-Santelises M, Sirsjö A, Wiklund NP (2006). Enhanced formation of nitric oxide in bladder carcinoma in situ and in BCG treated bladder cancer. Nitric Oxide.

[61] Andrade PM, Chade DC, Borra RC, Nascimento IP, Villanova FE, Leite LC, Andrade E, Srougi M (2010). The therapeutic potential of recombinant BCG expressing the antigen S1PT in the intravesical treatment of bladder cancer. Urol Oncol.

[62] Melo GD, Silva JE, Grano FG, Homem CG, Machado GF (2014). Compartmentalized gene expression of toll-like receptors 2, 4 and 9 in the brain and peripheral lymphoid organs during canine visceral leishmaniasis. Parasite Immunol.

[63] Benhar M, Stamler JS (2005). A central role for S-nitrosylation in apoptosis. Nat Cell Biol.

[64] Zeini M, Través PG, López-Fontal R, Pantoja C, Matheu A, Serrano M (2006). Specific contribution of p19(ARF) to nitric oxide-dependent apoptosis. J Immunol.

[65] Lim LY, Vidnovic N, Ellisen LW, Leong CO (2009). Mutant p53 mediates survival of breast cancer cells. Br J Cancer.

[66] Wang XW, Hussain SP, Huo TI, Wu CG, Forgues M, Hofseth LJ (2002). Molecular pathogenesis of human hepatocellular carcinoma. Toxicology.

[67] Wang C, Chen J (2003). Phosphorylation and hsp90 binding mediate heat shock stabilization of p53. J Biol Chem.

[68] Umansky V, Schirrmacher V (2001). Nitric oxide-induced apoptosis in tumor cells. Adv Cancer Res.

[69] Dameron KM1, Volpert OV, Tainsky MA, Bouck N (1994). Control of angiogenesis in fibroblasts by p53 regulation of thrombospondin-1. Science.

[70] Zhang ZG, Zhang L, Jiang Q, Zhang R, Davies K, Powers C, Bruggen N, Chopp M (2000). VEGF enhances angiogenesis and promotes blood-brain barrier leakage in the ischemic brain. J Clin Invest.

