# Supplementary material for: Increased toll-like receptors and p53 levels regulate apoptosis and angiogenesis in non-muscle invasive bladder cancer: mechanism of action of P-MAPA biological response modifier
Source: BMC Cancer. 2016 Jul 7;16:422. doi: 10.1186/s12885-016-2474-z (PMC4937612; doi:10.1186/s12885-016-2474-z)
Supplement: Additional file 1: Table S1. — Percentage of histopathological changes of the urinary bladder of rats from CONTROL, MNU, MNU-BCG and MNU-P-MAPA groups. (DOCX 61 kb) [file 12885_2016_2474_MOESM1_ESM.docx]

**Table S1:** Percentage of histopathological changes of the urinary bladder of rats from CONTROL, MNU, MNU-BCG and MNU-P-MAPA groups.

|  | **Groups** | | |  | |  |
| --- | --- | --- | --- | --- | --- | --- |
| **Histopathology** | **CONTROL**  *(n= 05)* | **MNU**  *(n= 05)* | **MNU-BCG**  *(n= 05)* | | **MNU-P-MAPA**  *(n= 05)* | |
| Normal | 05 (100%)* | - | - | | 03 (60%)* | |
| Flat Hyperplasia | - | - | - | | 01 (20%)* | |
| Papillary Hyperplasia | - | - | 01 (20%) | | 01 (20%) | |
| Low-grade Intraurothelial Neoplasia | - | - | 02 (40%)* | | - | |
| High-grade Intraurothelial Neoplasia – Flat Carcinoma *in situ* (pTis) | - | 01 (20%)* | - | | - | |
| Papillary Carcinoma *in situ* (pTa) | - | 02 (40%)* | 02 (40%) | | - | |
| Tumor invading mucosa or submucosa of the bladder wall (pT1) | - | 02 (40%)* | - | | - | |

^*^Statistical significance (test of proportion, *P*<0.0001)
