# Supplementary material for: Increased toll-like receptors and p53 levels regulate apoptosis and angiogenesis in non-muscle invasive bladder cancer: mechanism of action of P-MAPA biological response modifier
Source: BMC Cancer. 2016 Jul 7;16:422. doi: 10.1186/s12885-016-2474-z (PMC4937612; doi:10.1186/s12885-016-2474-z)
Supplement: Additional file 2: Table S2. — Semiquantitative analysis of immunolabelled antigens of the urinary bladder of rats in the different experimental groups. (DOCX 165 kb) [file 12885_2016_2474_MOESM2_ESM.docx]

**Table S2:** Semiquantitative analysis of immunolabelled antigens of the urinary bladder of rats in the different experimental groups.

|  |  | **Groups** | | | |
| --- | --- | --- | --- | --- | --- |
| **Antigens** | **CONTROL**  (*n= 05*) | | **MNU**  (*n= 05*) | **MNU-BCG**  (*n= 05*) | **MNU-P-MAPA**  (*n= 05*) |
| TLR2 | 2 (42.1%); 2+ | | 1 (15.8%); 1+ | 3 (87.4%); 3+ | 3 (95.1%); 3+ |
| MyD88 | 2 (22.5%); 2+ | | 1 (21.0%); 1+ | 3 (92.5%); 3+ | 3 (88.0%); 3+ |
| IKK-α | 1 (25.4%); 1+ | | 2 (47.4%); 2+ | 3 (88.9%); 3+ | 1 (22.3%); 1+ |
| NF-κB | 1 (28.3%); 1+ | | 3 (86.5%); 3+ | 2 (65.9%), 2+ | 1 (18.4%); 1+ |
| TNF-α | 1 (17.4%); 1+ | | 3 (84.%); 3+ | 3 (87.1%); 3+ | 1 (12.5%); 1+ |
| IL-6 | 1 (10.8%); 1+ | | 3 (85.1%); 3+ | 3 (91.7%); 3+ | 1 (10.0%); 1+ |
| TLR4 | 2 (45.4%); 2+ | | 1 (10.3%); 1+ | 3 (84.0%); 3+ | 3 (96.1%); 3+ |
| TRIF | 1 (11.8%); 1+ | | 1 (5.3%); 1+ | 2 (47.8%); 2+ | 3 (94.0%); 3+ |
| IRF-3 | 1 (7.9%); 1+ | | 1 (6.7%); 1+ | 2 (58.3%); 2+ | 3 (96.2%); 3+ |
| IFN-γ | 1 (22.4%); 1+ | | 1 (13.9%); 1+ | 2 (61.3%); 2+ | 3 (97.5%); 3+ |
| iNOS | 1 (10.0%); 1+ | | 1 (18.9%); 1+ | 2 (55.6%); 2+ | 3 (94.7%); 3+ |
| BAX | 1 (5.8%); 1+ | | 2 (57.1%); 2+ | 2 (62.3%); 2+ | 3 (85.4%); 3+ |

Percentage of labeled cells categorized into 4 scores as follows: 0, no immunoreactivity; 1, 1% – 35% positive urothelial cells; 2, 36% – 70% positive urothelial cells; 3, > 70% positive urothelial cells. Intensity of reactivity recorded as: weak (1+), moderate (2+) and intense (3+).
