# Supplementary figures and images for: Increased toll-like receptors and p53 levels regulate apoptosis and angiogenesis in non-muscle invasive bladder cancer: mechanism of action of P-MAPA biological response modifier
Source: BMC Cancer. 2016 Jul 7;16:422. doi: 10.1186/s12885-016-2474-z (PMC4937612; doi:10.1186/s12885-016-2474-z)

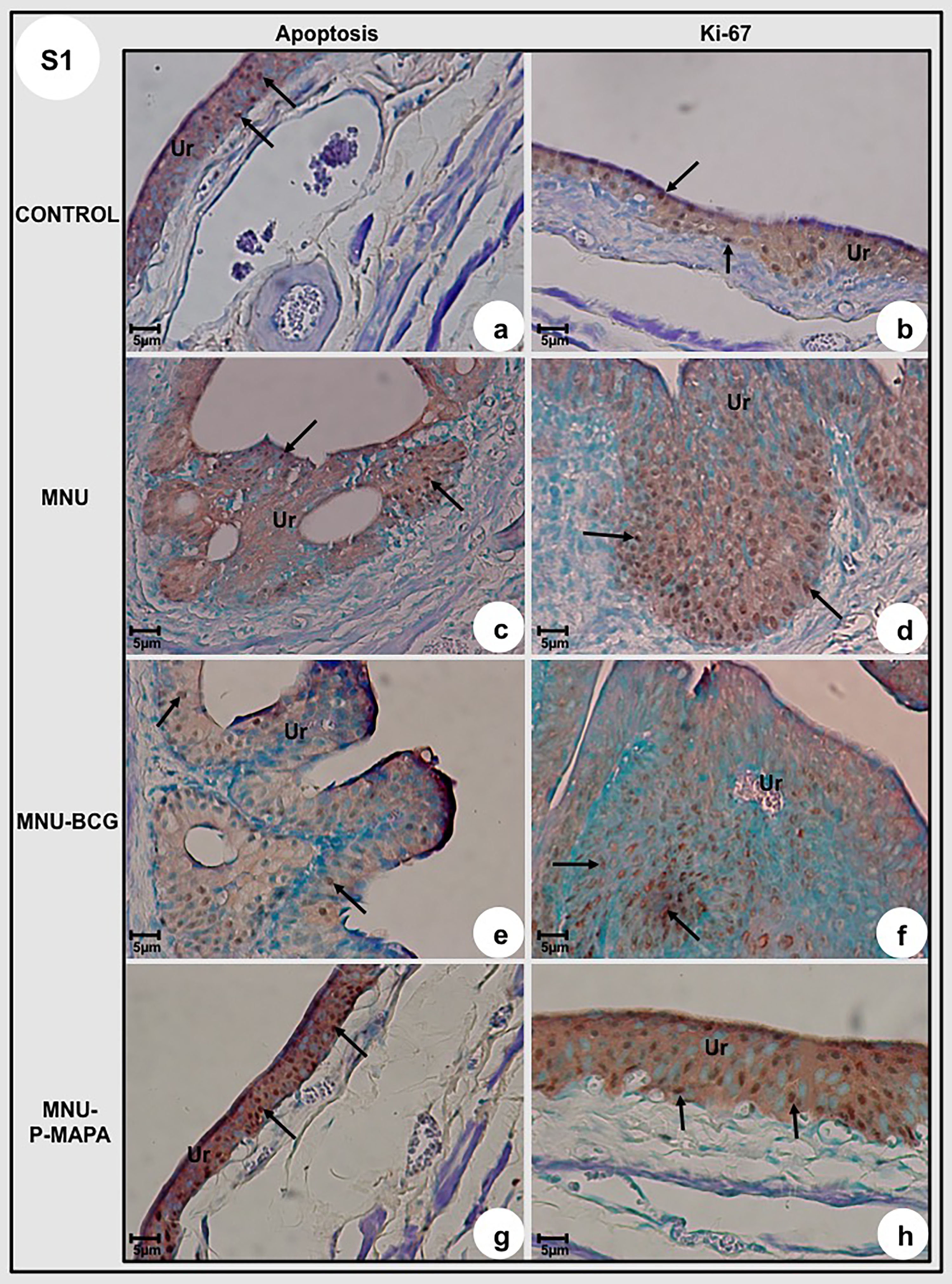

Supplement: Additional file 3: Figures S1a–S1h. — Immunolabelled Ki-67 intensities and detection of apoptosis in the urinary bladder from the CONTROL (a, b), MNU (c, d), MNU-BCG (e, f), and MNU-P-MAPA (g, h) groups. (a), (c), (e) and (g) DNA fragmentation (arrows) in the urothelium. (b), (d), (f) and (h) Ki-67 immunoreactivities (arrows) in the urothelium. a–h: Ur urothelium. (JPG 1261 kb) [file 12885_2016_2474_MOESM3_ESM.jpg]

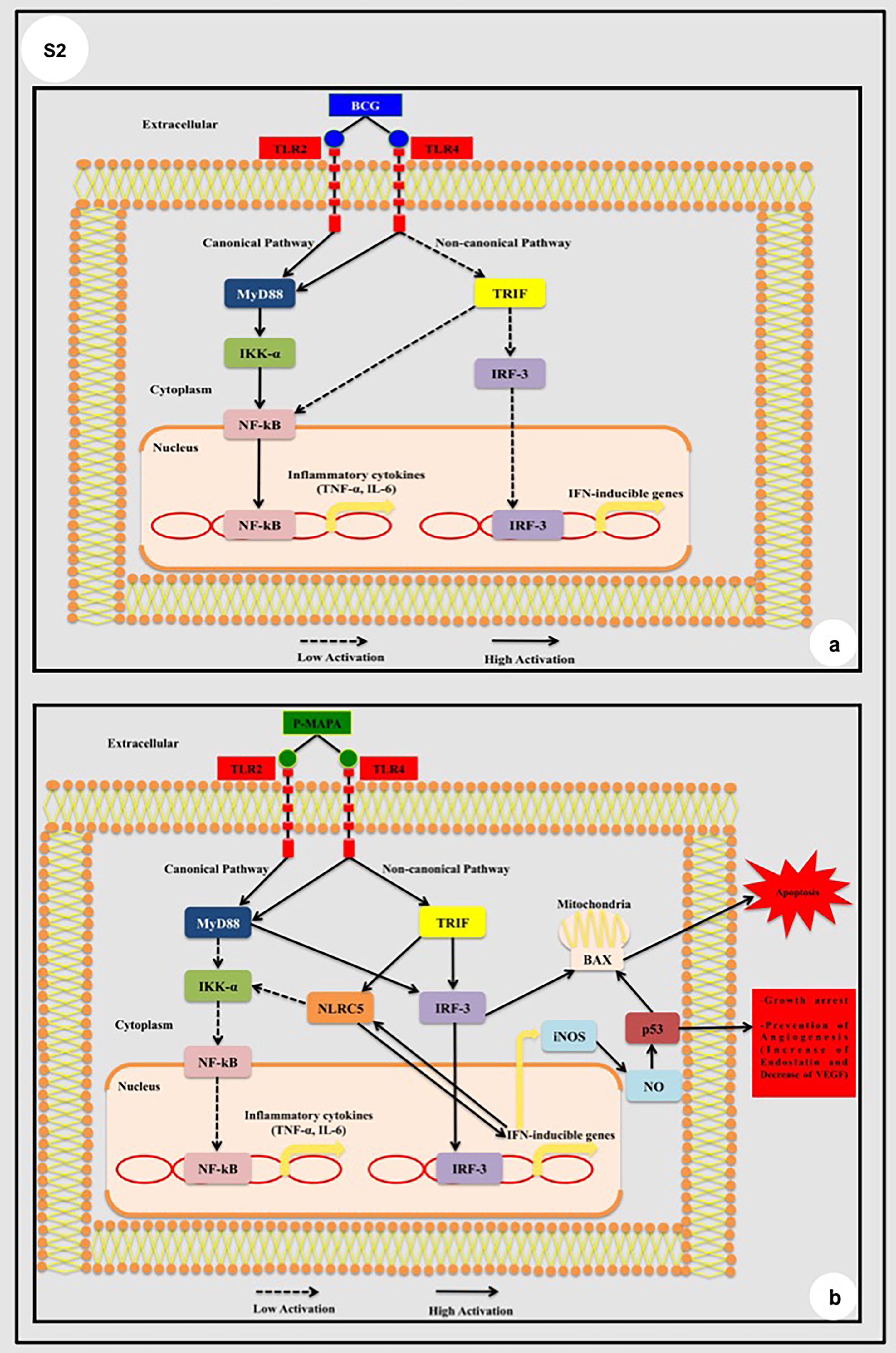

Supplement: Additional file 4: Figures S2a–S2b. — (a) Schematic representation of the mechanism of action of BCG involving TLRs signaling pathway. (b) Hypothetical mechanism of P-MAPA immunotherapy (Developed by Wagner José Fávaro and Farmabrasilis). (JPG 1048 kb) [file 12885_2016_2474_MOESM4_ESM.jpg]

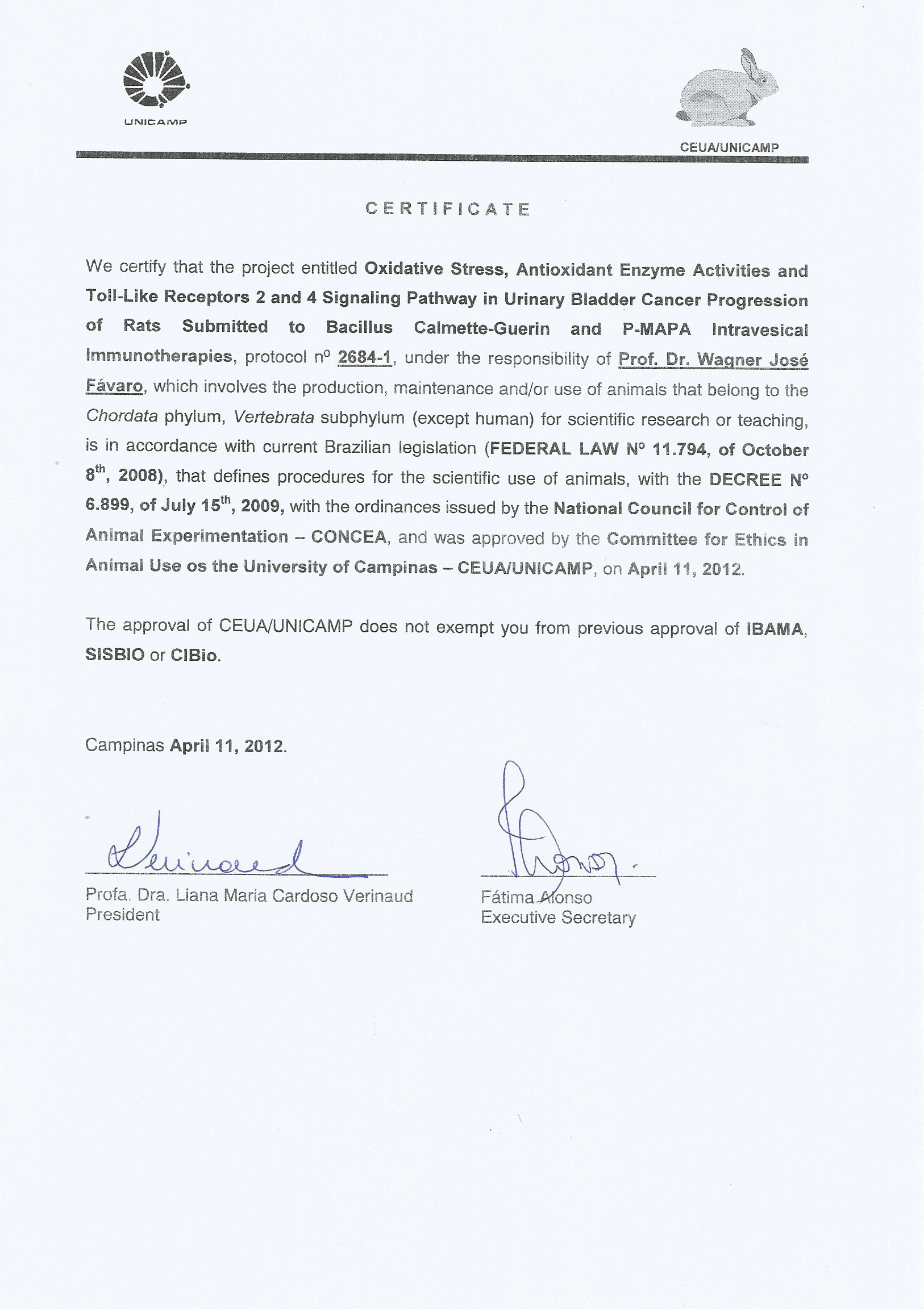

Supplement: Additional file 6: — Ethics Approval (JPG 1348 kb) [file 12885_2016_2474_MOESM6_ESM.jpg]
