# Supplementary material for: Increased toll-like receptors and p53 levels regulate apoptosis and angiogenesis in non-muscle invasive bladder cancer: mechanism of action of P-MAPA biological response modifier
Source: BMC Cancer. 2016 Jul 7;16:422. doi: 10.1186/s12885-016-2474-z (PMC4937612; doi:10.1186/s12885-016-2474-z)
Supplement: Additional file 5: — Availability of Data and Materials (DOCX 25 kb) [file 12885_2016_2474_MOESM5_ESM.docx]

| **Products** | **Description** | **Company** | **Link** |
| --- | --- | --- | --- |
| Ketamine | 60 mg/kg i.m | Ceva Animal Health Ltda, São Paulo, Brazil | <http://www.ceva.com.br/Produtos/Lista-de-Produtos/DOPALEN> |
| Xylazine | 5 mg/kg i.m | Ceva Animal Health Ltda, São Paulo, Brazil | <http://www.ceva.com.br/Produtos/Lista-de-Produtos/ANASEDAN> |
| n-methyl-n-nitrosourea (NMU) | 1.5 mg/kg intravesical | Sigma N1517 | <http://www.sigmaaldrich.com/catalog/> [product/sigma/n1517?lang=pt&region=BR](http://www.sigmaaldrich.com/catalog/product/sigma/n1517?lang=pt&region=BR) |
| Bacillus Calmette-Guérin (BCG) | 10^6^ CFU (40 mg) | Fundação Ataulpho de Paiva, Rio de Janeiro, Brazil | <http://www.fundacaoataulphodepaiva.com.br/vacina-bcg.html> |
| P-MAPA | 5 mg/kg | Farmabrasilis, Campinas, SP, Brazil | <http://www.farmabrasilis.org> |
| plastic polymer | Tissue Processing | Paraplast Plus, ST. Louis, MO, USA | <http://www.sigmaaldrich.com/catalog/>  [product/sigma/p3683?lang=pt&region=BR](http://www.sigmaaldrich.com/catalog/product/sigma/p3683?lang=pt&region=BR) |
| anti-TLR2 | (251110) | Abbiotec, San Diego, USA | <http://www.abbiotec.com/antibodies/tlr2-antibody> |
| anti-TLR4 | (251111) | Abbiotec, San Diego, USA | <http://www.abbiotec.com/antibodies/tlr4-antibody> |
| anti-MyD88 | (ab2064; 1:75) | Abcam, USA | <http://www.abcam.com/myd88-antibody-ab2064.html> |
| anti-IRF-3 | (ab25950; 1:150) | Abcam, USA | <http://www.abcam.com/irf3-antibody-ab25950.html> |
| anti-IKK-α | (ab38515; 1:100) | Abcam, USA | <http://www.abcam.com/ikk-alpha-phospho-t23-antibody-ab38515.html> |
| anti-BAX | (ab7977; 1:50) | Abcam, USA | <www.abcam.com/Bax-antibody-ab7977.pdf> |
| anti-NF-κB | (ab7970; 1:200), | Abcam, USA | <http://www.abcam.com/nf-kb-p65-antibody-chip-grade-ab7970-protocols.html> |
| anti-iNOS | (ab15323; 1:75) | Abcam, USA | <http://www.abcam.com/inos-antibody-ab15323.html> |
| anti-TNF-α | (ab6671; 1:150) | Abcam, USA | <http://www.abcam.com/tnf-alpha-antibody-ab6671.html> |
| anti-TRIF | (ab13810; 1:100) | Abcam, USA | <http://www.abcam.com/trif-antibody-ab13810.html> |
| anti-IL-6 | (ab6672; 1:150) | Abcam, USA | <http://www.abcam.com/il6-antibody-ab6672.html> |
| anti-IFN-γ | (507802; 1:50) | Biolegend, USA | <http://www.biolegend.com/purified-anti-rat-ifn-gamma-antibody-1548.html> |
| anti- Ki-67 | (NCL-Ki67-MM1; 1:50) | Novocastra; Newcastle, United Kingdom | <http://www.leicabiosystems.com/ihc-ish-fish/immunohistochemistry-ihc-antibodies-novocastra-reagents/primary-antibodies/details/product/ki67/> |
| AdvanceTM HRP kit |  | Dako Cytomation Inc., USA | <http://www.dako.com/br/ar49/p235369/prod_products.htm?setCountry=true&purl=ar49/p23536>  [prod_products.htm?undefined&submit=Accept%20country](http://www.dako.com/br/ar49/p235369/prod_products.htm?setCountry=true&purl=ar49/p235369/prod_products.htm?undefined&submit=Accept%20country) |
| RIPA lysis buffer | 50 μl/mg | EMD Millipore Corporation, Billerica, MA, USA | <https://www.emdmillipore.com/US/en/product/RIPA-Lysis-Buffer,-10X,MM_NF-20-188> |
| anti-p53 | (ab26; 1:250) | Abcam, USA | <www.abcam.com/p53-antibody-PAb-240-ab26.pdf> |
| anti-VEGF | (sc-53462; 1:300) | Santa Cruz Biotechnology, USA | <http://www.scbt.com/pt/datasheet-53462-vegf-vg-1-antibody.html> |
| anti-Endostatin | (ab64569; 1:250) | Abcam, USA | <http://www.abcam.com/endostatin-antibody-4i37-ab64569.html> |
| anti-NLRC5 | (ab105411; 1:300) | Abcam, USA | <http://www.abcam.com/nlrc5-antibody-ab105411.html> |
| HRP-conjugated antibodies | 1:3.000 in 1% BSA | Santa Cruz Biotechnology, Inc., Santa Cruz, CA, USA | <http://www.scbt.com/pt/datasheet-2030-goat-anti-rabbit-igg-hrp.html> |
| DNA fragmentation (TUNEL) by means of Terminal Deoxynucleotidyl Transferase (TdT) | Kit FragELTM DNA | Calbiochem, La Jolla, CA, USA | <http://www.emdmillipore.com/US/en/product/FragEL%E2%84%A2-DNA-Fragmentation-Detection-Kit,-Colorimetric---TdT-Enzyme,EMD_BIO-QIA33> |

**Samples Code for Histopathological, Immunochemistry and Western Blotting Analyses**

| **GROUPS** | **Samples Code for Histopathological and Immunohistochemistry** | **Samples Code for Western Blotting** |
| --- | --- | --- |
| CONTROL | WF 1 Control  WF 3 Control  WF 4 Control  WF 01/13 CT  WF 03/13 CT | - Pool 1,3,4 (Samples for animals 1, 2 and 3)  - Pool WF 01/13, WF 03/13 (Samples for animals 01 e 03/13) |
| MNU | WF 154/13 MNU  WF 155/13 MNU  WF 15/13 MNU  WF 16/13 MNU  WF 17/13 MNU | - Pool WF 154/13, WF 155/13 (Samples for animals 154 and 155/13)  - Pool WF 15/13, 16/13, 17/13 (Samples for animals 15, 16, 17/13) |
| MNU-BCG | WF 10 MNU-BCG  WF 11 MNU-BCG  WF 12 MNU-BCG  WF 198/13 MNU-BCG  WF 199/13 MNU-BCG | - Pool 10, 11, 12 (Samples for animals 10, 11 and 12)  - Pool 198, 199/13 (Samples for animals 198 and 199/13) |
| MNU-P-MAPA | WF 5 MNU-P-MAPA  WF 6 MNU-P-MAPA  WF 7 MNU-P-MAPA  WF 8 MNU-P-MAPA  WF 9 MNU-P-MAPA | - Pool 5, 6 (Samples for animals 5 and 6)  - Pool 7,8,9 (Samples for animals 7, 8 and 9) |
